# Solid-State Dynamic Behavior of the Boron–Nitrogen Bond Enables Mechanophotocatalysis of a [2 + 2]-Photocycloaddition

**DOI:** 10.1021/jacsau.6c00942

**Published:** 2026-08-21

**Authors:** Alaric Shaw, Peter F. Gilbert, Ivan Bondarenko, Kokoro K. Holcomb, Paulina M. Sittinger, Jesus Daniel Loya, Simon J. Teat, Nicholas S. Settineri, Gonzalo Campillo-Alvarado

**Affiliations:** † Department of Chemistry, 6686 Reed College, Portland, Oregon 97202-8199, United States; ‡ Institut für Chemie und Biochemie, Freie Universität Berlin, Arnimallee 22, 14195 Berlin, Germany; § Department of Chemistry, Amherst College, Amherst, Massachusetts 01002, United States; ∥ Advanced Light Source, Lawrence Berkeley National Laboratory, Berkeley, California 94720, United States; ⊥ Department of Chemistry, 1438 University of California, Berkeley, Berkeley, California 94720-1460, United States

**Keywords:** mechanochemistry, boron coordination, [2 + 2]-photocycloaddition, self-assembly, boronic ester, catalysis

## Abstract

We report reversible B←N coordinate bond formation and cleavage in the organic solid state. The dynamic behavior was probed using the [2 + 2]-photodimerization of 4-(1-naphthylvinyl)­pyridine (**4-nvp**) as a chemical reporter under concurrent mechanochemical vortex grinding and ultraviolet light-emitting diode (UV-LED) irradiation. Phenylboronic acid catechol ester (**pbe**) templates the regio- and stereoselective formation of 1,3-bis­(4-pyridyl)-2,4-bis­(1-naphthyl)­cyclobutane (**pncb**), and the reaction proceeds efficiently even when **pbe** is used in substoichiometric amounts (50%, 30% and 5% mol). The catalytic turnover demonstrates that B←N bonds can break and reform dynamically under mechanical stress in the organic solid state, establishing B←N coordination as a design element for responsive molecular materials.

## Graphical abstract

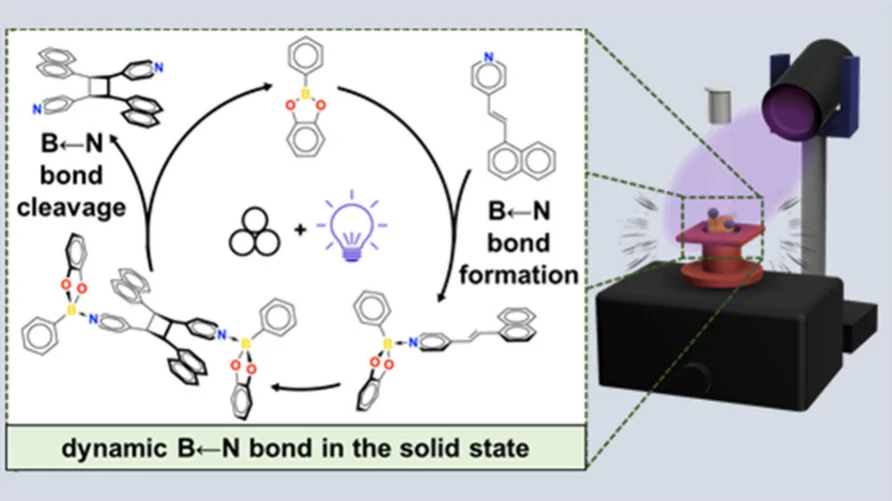

Dynamic chemical bonds (DCBs) have emerged as a powerful design element in materials chemistry and nanoscience in recent years.^1^ By undergoing reversible cleavage and recombination of covalent or noncovalent bonds, DCBs allow molecular materials to adapt dynamically to external physical and chemical stimuli (e.g., mechanical stress, heat, light).^1,2^ As a result of the structural plasticity, bulk materials with DCBs often exhibit multireversible properties, including stimuli-responsiveness, shape-memory behavior, and autonomous self-healing.^3–8^ In this context, boronate ester linkages have been used to construct resilient self-healing solids and gels, primarily exploiting the reversibility of covalent B–O bonds via transesterification and metathesis.^9–11^

The coordinate (dative) B←N bond shares a similar potential for reversible reactivity,^12^ enabling the formation of diverse discrete and extended molecular architectures, including polymers, cages and macrocycles.^13–20^ A recent report demonstrated the solution-based interconversion of *N*-based aromatic heterocycles with an organoboronic ester to generate a dynamic library of B←N adducts.^21^ While the dynamic properties of B←N bonds are widely documented in solution and soft matter, this dynamic behavior has remained extremely rare and underexplored in the solid state, with only a few instances involving photoactive organoboroxine systems.^22–24^

We recently demonstrated that mechanochemistry can effectively direct the reaction outcome between boronic acids with *N*-donors, selectively yielding either boroxine adducts or multicomponent cocrystals.^25^ Inspired by the mechanochemical control, we envisaged that mechanical force could act as a direct stimulus to break and reform B←N bonds in the crystalline solid state.^26,27^

Herein, we demonstrate the B←N coordinate bond to exhibit dynamic behavior in the solid state using mechanochemistry. To demonstrate the dynamic exchange, we used a [2 + 2]-photocycloaddition as a chemical reporter,^28^ achieving solid-state supramolecular catalysis through concurrent mechanochemical vortex grinding and ultraviolet light-emitting diode (UV-LED) irradiation (Scheme 1A,B).^29–33^ Specifically, vortex grinding phenylboronic acid catechol ester (**pbe**) with *N*-donor 4-(1-naphthylvinyl)­pyridine (**4-nvp**) directs the near-quantitative (95–99% conversion) formation of the bis-boron head-to-tail (HT) adduct of *rctt*-1,3-bis­(4-pyridyl)-2,4-bis­(1-naphthyl)­cyclobutane [**2**(**pbe**)·(**HT-pncb**)]. Remarkably, because the B←N bond can dynamically break and reform under mechanical force, the reaction proceeds catalytically (Scheme 1C). By employing substoichiometric loadings of 50%, 30%, and even 5% mol of **pbe**, near-quantitative yields (ca. 95%) of the photoproduct are achieved in less than 2 h. The efficient catalytic turnover in the [2 + 2]-photodimerization demonstrates that the B←N bond is actively cleaving and reforming in the solid state and serves as a direct reporter.

**Scheme 1:**
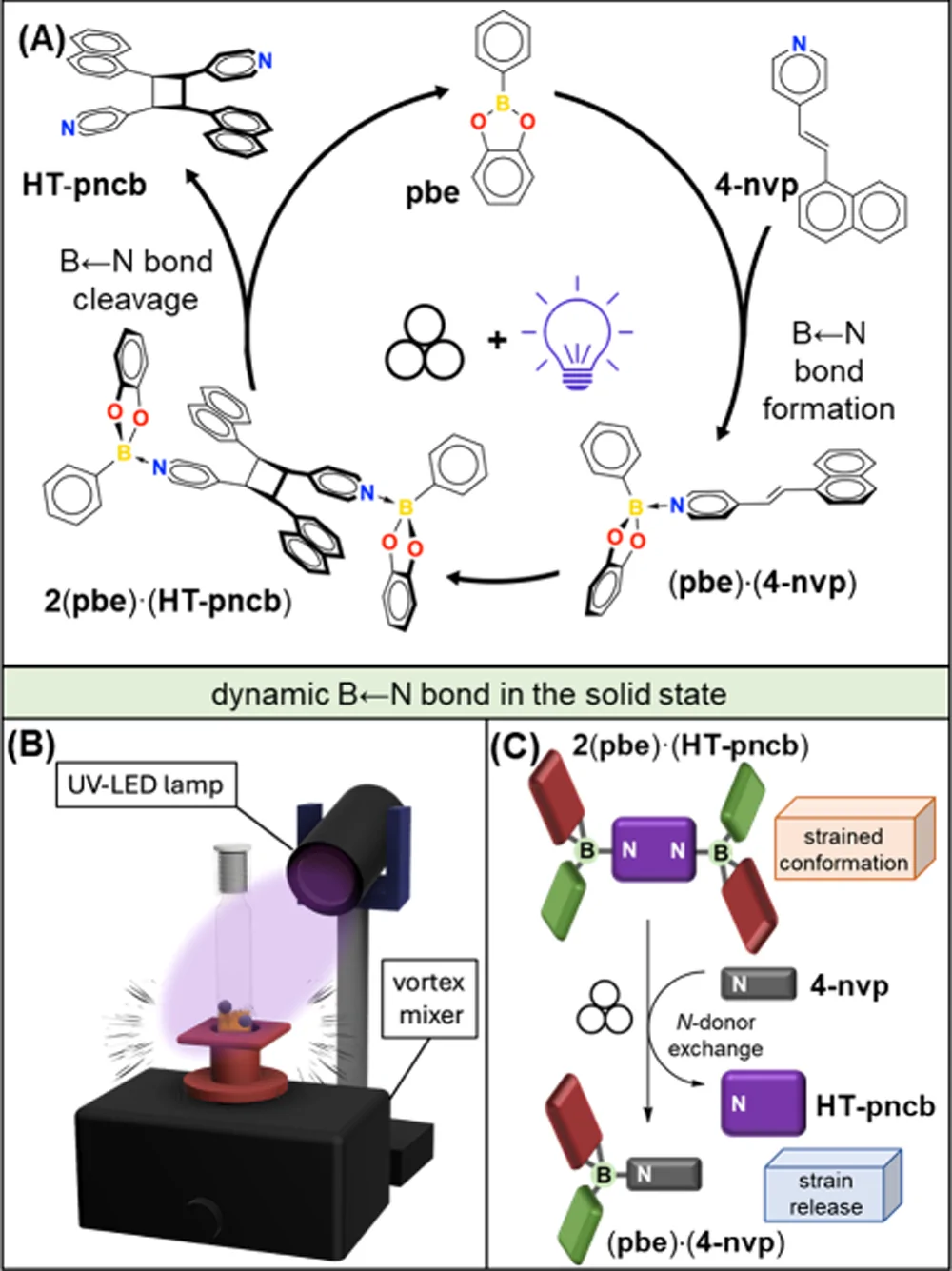
Mechanophotocatalysis Enabled by Dynamic B←N Bonds in the Organic Solid State: (A) Catalytic Cycle of a [2 + 2]-Photodimerization Enabled by B←N Bond Formation and Cleavage; (B) Setup for Concurrent Mechanochemistry and UV-LED Irradiation of Powder Samples; (C) Catalytic Turnover Is Enabled by Mechanochemical Cleavage and Formation of B←N Bonds in the Solid State

Powder X-ray diffraction (PXRD) monitoring supports a mechanism in which mechanochemical cleavage of the strained B←N adduct releases **HT-pncb**, thereby enabling catalytic turnover. To the best of our knowledge, this work represents the first example and application of dynamic B←N bond cleavage and reformation using mechanochemistry in the organic solid state.

The photoactive solid that supports the photomechanochemical reaction is the B←N adduct (**pbe**)·(**4-nvp**). Single crystals of (**pbe**)·(**4-nvp**) were obtained by combining **pbe** (23 mg, 0.12 mmol) with **4-nvp** (27 mg, 0.12 mmol) in warm acetonitrile (10 mL). The mixture was then split into two scintillation vials and allowed to slowly evaporate over 3 days.

Single-crystal X-ray diffraction (SCXRD) using a synchrotron radiation source,^34^ which was used to minimize *in situ* photoreaction during data collection, revealed the highly reactive adduct (**pbe**)·(**4-nvp**) to crystallize in the triclinic space group *P*-1. The asymmetric unit comprises a single **pbe** ester coordinated to **4-nvp** *via* a dative B←N bond (1.624(1) Å), generating a T-shaped adduct. The tetrahedral character (THC) of the B atom (80.5%) is slightly larger, and the B←N bond length is shorter compared to those of similar monoboronic esters with *N*-donors, indicating a stronger interaction.^35–39^ In the **4-nvp** ligand, the naphthyl and pyridyl rings lie approximately coplanar (twist angle: 11.3°). Adjacent adducts assemble into discrete head-to-tail dimers sustained *via* face-to-face [π···π] interactions between overlapping pyridyl and naphthyl rings (Figure 1A).

The dimers are further sustained by auxiliary [C–H···π] contacts between neighboring naphthyl and catecholate rings (Figure 1B). Importantly, the carbon–carbon double (C=C) bonds in dimers are aligned in parallel at a distance of 3.6362(11) Å. The geometry satisfies Schmidt’s topochemical criteria for a [2 + 2]-photodimerization. Neighboring dimers pack through additional face-to-face [π···π] and [C–H···π] contacts, forming layers in the *ab*-plane (Figure 1C).

The (**pbe**)·(**4-nvp**) crystals are highly photoactive. Specifically, when data collection was attempted using a traditional home-source X-ray diffractometer, the crystal underwent partial, spontaneous transformation into the photoproduct. To drive the reaction to completion, crystals (**pbe**)·(**4-nvp**) were exposed to a 36 W UV lamp (λ = 400 nm, NuLink) for 10 min, which resulted in a quantitative single-crystal-to-single-crystal (SCSC) transformation^40–42^ to the bis-boron adduct 2­(**pbe**)·(**HT-pncb**).

SCXRD analysis of the irradiated crystal confirmed that the [2 + 2]-photodimerization proceeds regio- and stereoselectively, and in quantitative yield. The 2­(**pbe**)·(**HT-pncb**) structure is comprised of two **pbe** units acting as coordination “hinges” anchored to a central **HT-pncb** unit core *via* B←N bonds (Figure 2A). Following the photoreaction, the B←N bond distance increases to 1.647(3) Å, while the THC of the boron atom decreases to 77.4%, indicating a weakened interaction. The structural distortion is attributed to increased steric hindrance and conformational strain imposed by the reoriented naphthyl groups resulting from cyclobutane formation around the boron coordination environment. Moreover, the distance between neighboring naphthyl groups contracts from 4.2316(6) Å in (**pbe**)·(**4-nvp**) to 4.217(1) Å in 2­(**pbe**)·(**HT-pncb**), resulting in a shortened π-stacking reminiscent of [2 + 2]-photodimerizations in metal complexes.^43^ A related example of strain induced B←N bond cleavage was recently observed in an intramolecular [2 + 2]-photodimerization of a boroxine crystal, where bond breakage enabled strain release and macroscopic photosalient effects.^24^ Fourier transform infrared (FTIR) spectroscopy further supports the mechanophotochemical transformation (Figure S6.1, Supporting Information (SI)). Upon UV-LED irradiation and grinding, changes near ca. 1600 cm^–1^ and the appearance of a new band near ca. 1200 cm^–1^ are consistent with alkene consumption and cyclobutane C–C–H bending modes, respectively.^44^

**Figure 2:**
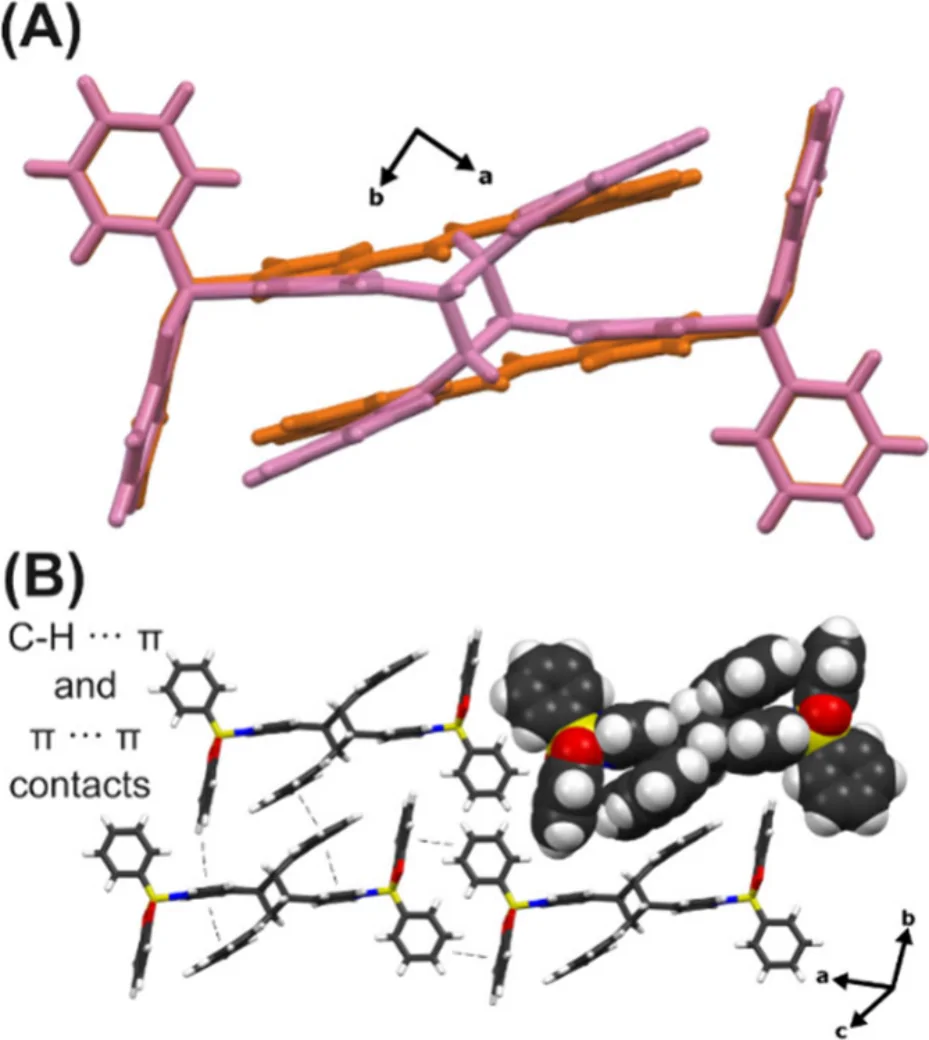
X-ray structure of 2­(**be**)·(**HT-pncb**): (A) Overlay with crystal structure of (**pbe**)·(**4-nvp**) showing SCSC transformation. (B) Face-to-face [π···π] and [C–H···π] contacts enable close-packing of photoproducts.

To evaluate electronic changes in the structures, electrostatic potential (ESP) maps were calculated using single-point density functional theory (DFT) with the ωB97X-D hybrid functional and the cc-pVTZ basis set (Figure S5.1, SI).^45,46^ Upon coordination to the electron-withdrawing **pbe** ester, the pyridyl rings in both (**pbe**)·(**4-nvp**) and 2­(**pbe**)·(**HT-pncb**) become highly electron-deficient. The induced charge complementarity with electron-rich regions results in an efficient network of supporting [C–H···π] and [π···π] forces throughout the crystal lattice, as seen in host–guest systems.^20,47–49^

The unusually fast reactivity of (**pbe**)·(**4-nvp**) crystals prompted us to investigate whether this transformation could be achieved directly from bulk starting materials *via* simultaneous mechanochemical vortex-grinding and UV-LED irradiation (Kessil PR160L, 456 nm, 55W max.).^31,50^ Specifically, **pbe** (115 mg, 0.59 mmol) and **4-nvp** (135 mg, 0. 59 mmol), along with 5 drops of acetonitrile, were placed in an Ace glass pressure vessel (15 mL) equipped with one stainless steel ball (7 mm diameter) (see Figure S1.1). Prior to starting UV-LED irradiation, a sample was coground to ensure homogeneity and studied by ^1^H NMR spectroscopy and PXRD (time 0 min). The vessel was secured in a vortex mixer and subjected to continuous grinding and concurrent UV-LED irradiation. Remarkably, ^1^H NMR spectra confirmed the near-quantitative (95%) formation of 2­(**pbe**)·(**HT-pncb**) after 120 min of continuous photomechanochemical processing (Figure 4A). The reaction progress was monitored by the progressive disappearance of the α-pyridyl proton resonance (Ha) of (**pbe**)·(**4-nvp**) at 8.60 ppm with the corresponding emergence of the α-pyridyl proton for the photoproduct 2­(**pbe**)·(**HT-pncb**) (Ha′) at 8.09 ppm, in addition to the disappereance of ethylene protons at 8.38 and 7.31 ppm, and appereance of the cyclobutane signal at 5.20 ppm (Figure 3A).^51^ The ^1^H NMR spectral transformation is in agreement with reported [2 + 2]-photodimerizations of **4-nvp**.^22,23,52–54^

**Figure 3:**
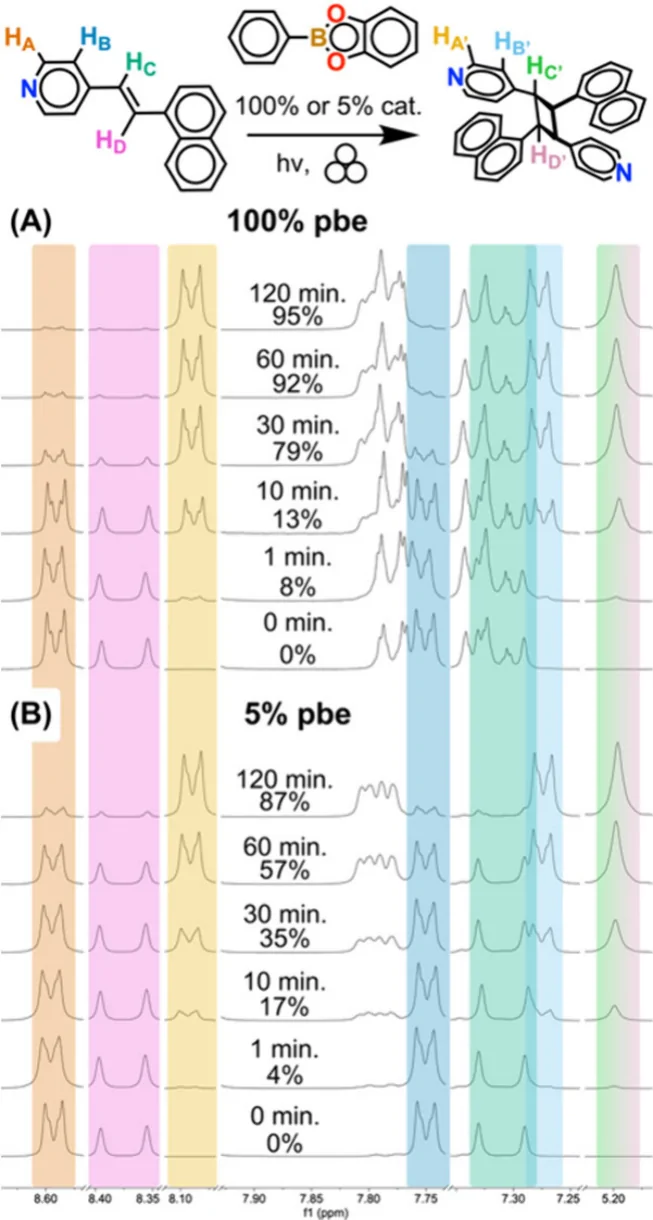
^1^H NMR spectra (400 MHz, DMSO-*d*_6_) showing mechanophotocatalysis using **pbe** (100% and 5% mol catalyst) and **4-nvp** to afford **HT-pncb**.

We hypothesized that if the B←N bond could dynamically break and reform under mechanical stress, the solid-state transformation would proceed catalytically, as previously reported for hydrogen and halogen bonding.^29–31,55^ To test our hypothesis, **4-nvp** was ground with substoichiometric **pbe** catalyst loadings of 50, 30, and 5% mol. When 50 and 30% mol catalyst were used, mechanophotocatalysis afforded near-quantitative yields of 97 and 99%, respectively, after 120 min (Figures S4.1–S4.6, SI). Remarkably, lowering the catalyst loading to just 5% mol still achieved an 87% conversion to **HT-pncb** within 120 min (Figure 3B). Prolonged grinding and UV-LED irradiation afforded a 99% conversion yield after a total of 180 min. To the best of our knowledge, this represents the lowest catalyst loading and shortest reaction time reported to date for an efficient solid-state supramolecular [2 + 2]-photodimerization.^56^ The catalytic turnover confirms the dynamic, reversible nature of the B←N bond under mechanical stress, showcasing the potential of using DCBs for small-molecule mechanophotocatalysis.

While ^1^H NMR spectroscopy confirms the near-quantitative formation of the cyclobutane core, the coordinate adduct dissociates in solution (DMSO-d_6_). To gain insight into the bound versus unbound states during the solid-state transformation, PXRD was utilized to monitor the equimolar (100%) and catalytic (50, 30 and 5% mol) reactions (Figure 4 and Figures S3.1–S3.5, SI)).

**Figure 4:**
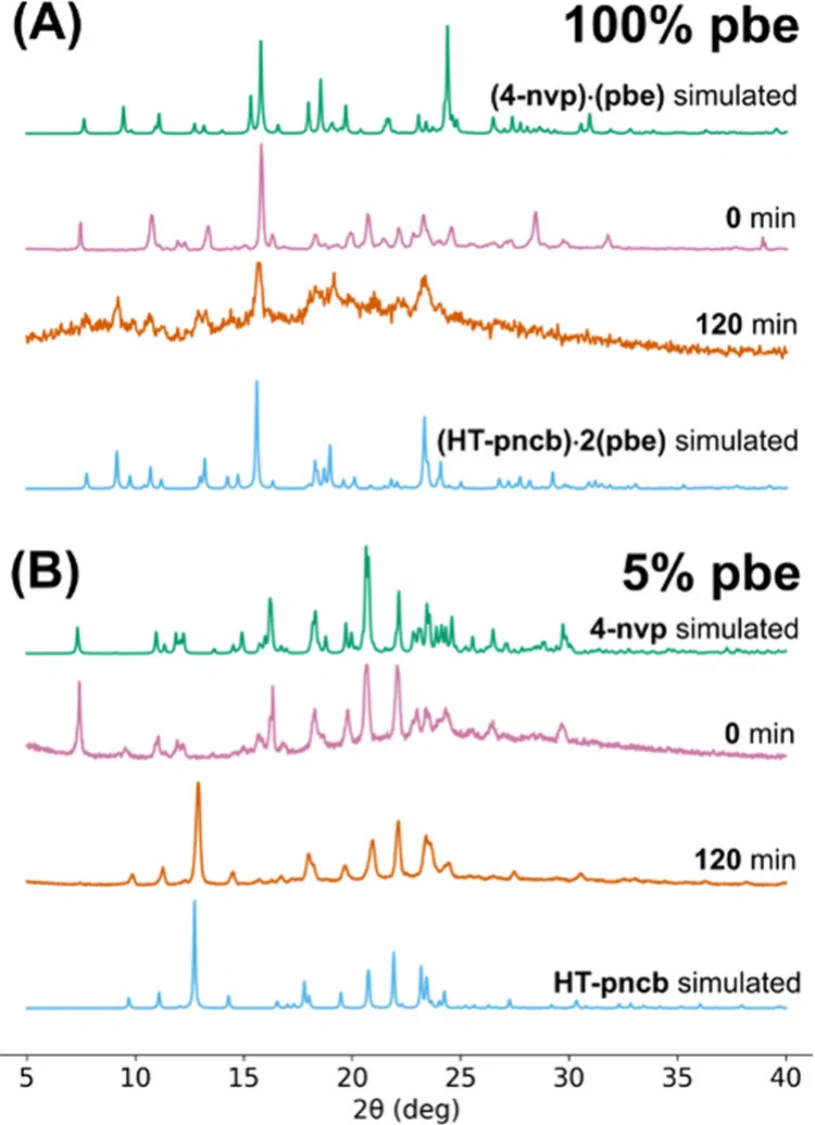
PXRD monitoring of the patterns of solid-state photomechanochemical reaction: (A) Stoichiometric reaction with 100% mol cat. **pbe**, affording the bound adduct 2­(**pbe**)·(**HT-pncb**). (B) Catalytic reaction with 5% mol cat. **pbe**, affording the unbound photoproduct **HT-pncb**. In both cases, the 0 min time point represents the preirradiation state, recorded after 10 s of preliminary grinding. Simulated patterns from SCXRD data (green and blue traces) of **4-nvp**, **HT-pncb**, (**pbe**)·(**4-nvp**), 2­(**be**)·(**HT-pncb**) are shown for comparison.

Specifically, PXRD revealed that a brief 10-s grinding period without UV irradiation (designated as time 0 min) readily formed the (**pbe**)·(**4-nvp**) adduct. This is confirmed by the appearance of prominent peaks at 7.4, 10.7, and 15.8° 2θ that match the simulated PXRD pattern from the SCXRD data. Following 120 min of simultaneous UV-LED exposure and vortex grinding, the bulk pattern shifted to match the simulated 2­(**pbe**)·(**HT-pncb**) structure, as observed by prominent peaks at 9.1, 15.6, and 24.1° 2θ, confirming the formation of the fully bound photoproduct.

Importantly, mechanophotocatalysis with substoichiometric catalyst loadings (50, 30, and 5%) exhibited distinct solid-state behavior. After 120 min of UV-LED exposure, the PXRD patterns for all three catalytic reactions showed a prominent diagnostic peak at 12.8° 2θ, corresponding to the unbound photoproduct, **HT-pncb** (CCDC 1825521).^57^ In the 5% experiment, minor peaks were observed at 7.5 and 16.1° 2θ, corresponding to unreacted **4-nvp** (CCDC 2334116),^58^ which is consistent with the 87% conversion yield. Notably, the catalytic reactions exhibit sharper PXRD peaks than the stoichiometric reaction (Figure S3.5, SI), suggesting that dynamic B←N cleavage and reformation may provide a pathway for releasing strain generated by simultaneous grinding and photodimerization, similarly to dynamic covalent organic frameworks and polymers.^59,60^ The observations provide evidence that the mechanophotocatalytic cycle involves a mechanical turnover step comprising cleavage of the dynamic B←N bond, releasing **HT-pncb** and allowing **pbe** to coordinate fresh **4-nvp** molecules.^56^ Furthermore, the high reaction yields achieved under substoichiometric conditions (i.e., the bulk matrix contains an excess of free **4-nvp**) indicate that **pbe** is not irreversibly trapped by the photoproduct **HT-pncb**, thereby sustaining catalytic turnover without significant product inhibition.

The PXRD and SCXRD results support a mechanism in which mechanical stress facilitates cleavage of the strained B←N adduct after photodimerization, releasing **HT-pncb** and allowing **pbe** to coordinate free **4-nvp** molecules during catalytic turnover. The release of unbound **HT-pncb** under catalytic conditions is consistent with the more sterically hindered B←N coordination environment generated upon cyclobutane formation.^24^

In summary, we have demonstrated the reversible behavior of the B←N bond facilitated by mechanochemistry in the organic solid state. Using a [2 + 2]-photodimerization as a chemical reporter, we show that B←N bond formation and cleavage can enable catalytic turnover via the release of **HT-pncb** and recoordination of **pbe** to free **4-nvp** molecules. The work paves the way for future advances using dynamic B←N coordination^22,23^ as a solid-state design element for mechanochemical reactivity and provides a platform for the development of smart molecular materials for sensing and recognition applications.^61–63^

## Supplementary Material



## Notes

Experimental information, NMR spectra, PXRD patterns, molecular modeling data, and additional SCXRD data (PDF)
