# Supplementary material for: Solid-State Dynamic Behavior of the Boron–Nitrogen Bond Enables Mechanophotocatalysis of a [2 + 2]-Photocycloaddition
Source: JACS Au. 2026 Aug 21;6(9):4897–902. doi: 10.1021/jacsau.6c00942 (PMC13625742; doi:10.1021/jacsau.6c00942)
Supplement: Supplementary file 1 [file au6c00942_si_001.pdf]

Supporting Information (SI)

Solid-State Dynamic Behavior of the Boron–Nitrogen Bond Enables Mechanophotocatalysis of a [2 + 2]-Photocycloaddition

Alaric Shaw,[a] Peter F. Gilbert,[a] Ivan Bondarenko,[a] Kokoro K. Holcomb,[a] Paulina M. Sittinger,[a,b] Jesus Daniel Loya,[a,c] Simon J. Teat,[d] Nicholas S. Settineri,[d,e] and Gonzalo Campillo-Alvarado[a,*]

[a]Department of Chemistry, Reed College, Portland, OR, 97202-8199, United States

[b]Institut für Chemie und Biochemie, Freie Universität Berlin, Arnimallee 22, 14195 Berlin, Germany

[c]Department of Chemistry, Amherst College, Amherst, MA, 01002, United States

[d]Advanced Light Source, Lawrence Berkeley National Laboratory, Berkeley, CA, 94720, United States

[e]Department of Chemistry, University of California, Berkeley, Berkeley, California, 94720-1460, United States

Table of Contents

S1) Experimental information

S2) Single-crystal X-ray diffraction data

S3) Powder X-ray diffraction data

S4) NMR spectroscopy data

S5) Molecular modeling data

S6) Fourier-transform infrared data

S7) Thermogravimetric analysis data

S1. Experimental information

Materials and Methods:

Acetonitrile was purchased from Sigma-Aldritch, 4-(1-napthylvinyl)pyridine (**4-nvp**) was purchased from TCI America, catechol was purchased from Combi-Blocks, and phenylboronic acid was purchased from AmBeed. All chemicals were used as received without further purification.

SCXRD data for 2(**pbe**)·(**HT-pncb**) were collected on a Bruker D8 diffractometer equipped with a PHOTON II CPAD detector with synchrotron radiation (λ = 0.7288 Å) on beamline 12.2.1 at Advanced Light Source. The intensity data with synchrotron radiation were collected in APEX3,[1] integration and corrections were applied with SAINT v8.40a,[2] and absorption and other corrections were made using SADABS 2016/2.[3] Dispersion corrections appropriate for this wavelength were calculated using the Brennan method in XDISP[4] within WinGX.[5] The structures were solved with a dual space method with SHELXT 2018/2[6] and refined using SHELXL 2019/2.[7] All non-hydrogen atoms were refined anisotropically. Hydrogen atoms were placed geometrically on the carbon atoms and refined with a riding model. SCXRD data for 2(**pbe**)·(**HT-pncb**) were collected on a Rigaku XtaLAB Mini II diffractometer with a CCD area detector (λ = 0.71073 Å, graphite monochromator). The structures were solved and refined using CrysAlis and OLEX2 equipped with the SHELXT[6] and SHELXL[7] packages for crystal structure refinement. All non-hydrogen atoms were refined anisotropically. Hydrogen atoms were placed geometrically on the carbon atoms and refined with a riding model.

The UV-LED used was a Kessel PR160L-456 with a matrix LED emitting 456 nm light with a power maximum of 55 W. According to the manufacturer's specifications, at 10 cm from the lamp, this produces approximately 10 mW/cm^2 of UV light.

Fourier-transform infrared (FT-IR) spectra were recorded using a Thermo Fisher Scientific iS5 IR spectrometer from 600 to 4000 cm–1 using a diamond attenuated total reflectance (ATR) accessory.

Thermogravimetric analysis (TGA) data were collected on a Perkin-Elmer Pyris 9 instrument using a heating rate of 5 ^{o}C/min under nitrogen gas.

^{1}H NMR spectra were collected using a Bruker Ascend Evo 400 MHz spectrometer with DMSO-d_6 and tetramethylsilane as internal standards. NMR data were processed with the Mnova suite.

PXRD data were collected on a Scintag XDS-2000 diffractometer using CuKα1 radiation (λ = 1.5418° Å). The samples were mounted and collected on glass slides, typically in the range of 5–40° two-theta (scan type: step size: 0.02°, rate: 3 deg/min, continuous scan mode). The equipment was operated at 40 kV and 30 mA, and the data were collected at room temperature.

Single crystals of (**pbe**)·(**4-nvp**) were grown by combining **pbe** (23.1 mg, 0.12 mmol) and **4-nvp** (27.0 mg, 0.12 mmol), then lightly grinding them into a homogenous powder in an agate mortar and pestle. The resulting solid was then split between two 20 mL scintillation vials, and both were dissolved in 5 mL of warm acetonitrile with sonication to ensure full dissolution. Both vials were wrapped in aluminum foil to prevent spontaneous photoreactions. Crystals of (**pbe**)·(**4-nvp**) grew as large opaque yellow prisms. Crystals of 2(**pbe**)·(**HT-pncb**) were created by exposing crystals of (**pbe**)·(**4-nvp**) to a mild 36 W UV nail lamp (λ = 400 nm, NuLink), which after 10 minutes, resulted in a quantitative single-crystal-to-single-crystal conversion to crystals of 2(**pbe**)·(**HT-pncb**).

Preparation of phenyl boronic acid catechol ester (pbe):

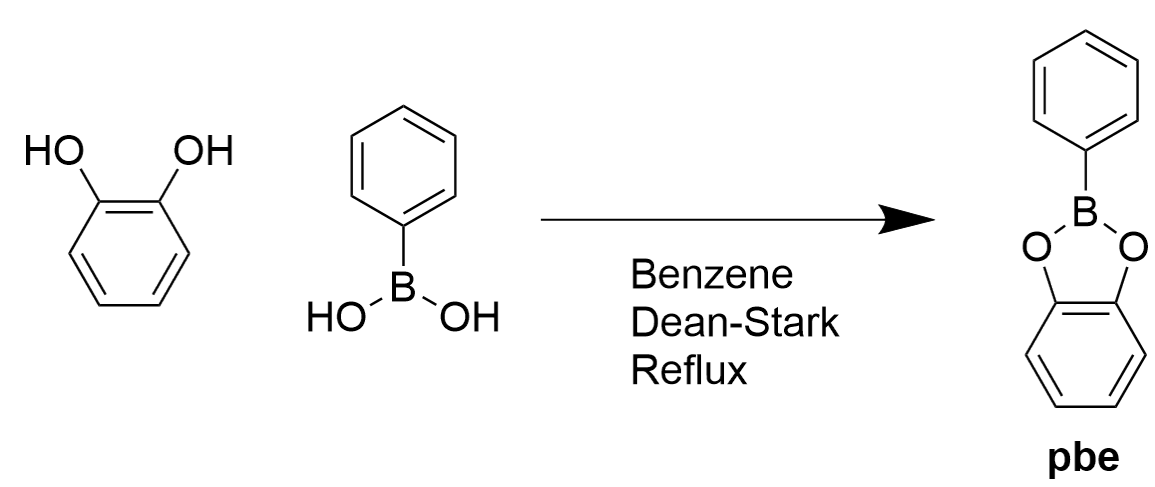

Phenylboronic acid (3.0 g, 24.6 mmol) was combined with catechol (2.7 g, 24.5 mmol) in a 250 mL round-bottom flask. 200 mL of benzene was added to the round-bottom flask, as well as 20 drops of methanol. The solution was sonicated until all components were fully dissolved. The round-bottom flask was equipped with a Dean-Stark apparatus and a condenser, then refluxed under stirring. The temperature was increased until liquid began collecting in the Dean-Stark trap, and the reaction was then left to run overnight. The remaining benzene in the round-bottom flask was reduced in *vacuo*, and **pbe** was collected as a white powder and used without further purification.

General Procedure for Mechanophotocatalysis:

250 mg (1.08 mmol) of **4-nvp** was combined with the corresponding molar ratio of **pbe** such that a 1:1, 1:2, 3:10, 1:10, or 1:20 ratio was achieved. The dry mixture was then lightly ground to ensure homogeneity. NMR and PXRD samples were collected from this mixture. 250 mg of the mixture was loaded into a 15 mL Ace glass pressure vessel along with 5 drops of acetonitrile and one stainless steel ball bearing. The reaction vessel was then placed in a vortex mixer and concurrently exposed to ultraviolet light from a UV-LED (Kessil PR160L, 456nm, 55W max.). The grinding process continued for two hours, with short breaks to take NMR samples at 1, 10, 30, 60, and 120 minutes, as well as PXRD samples at 1 and 120 minutes. At each time point, a metal spatula was used to scrape any compound stuck to the inner walls of the reaction vessel.

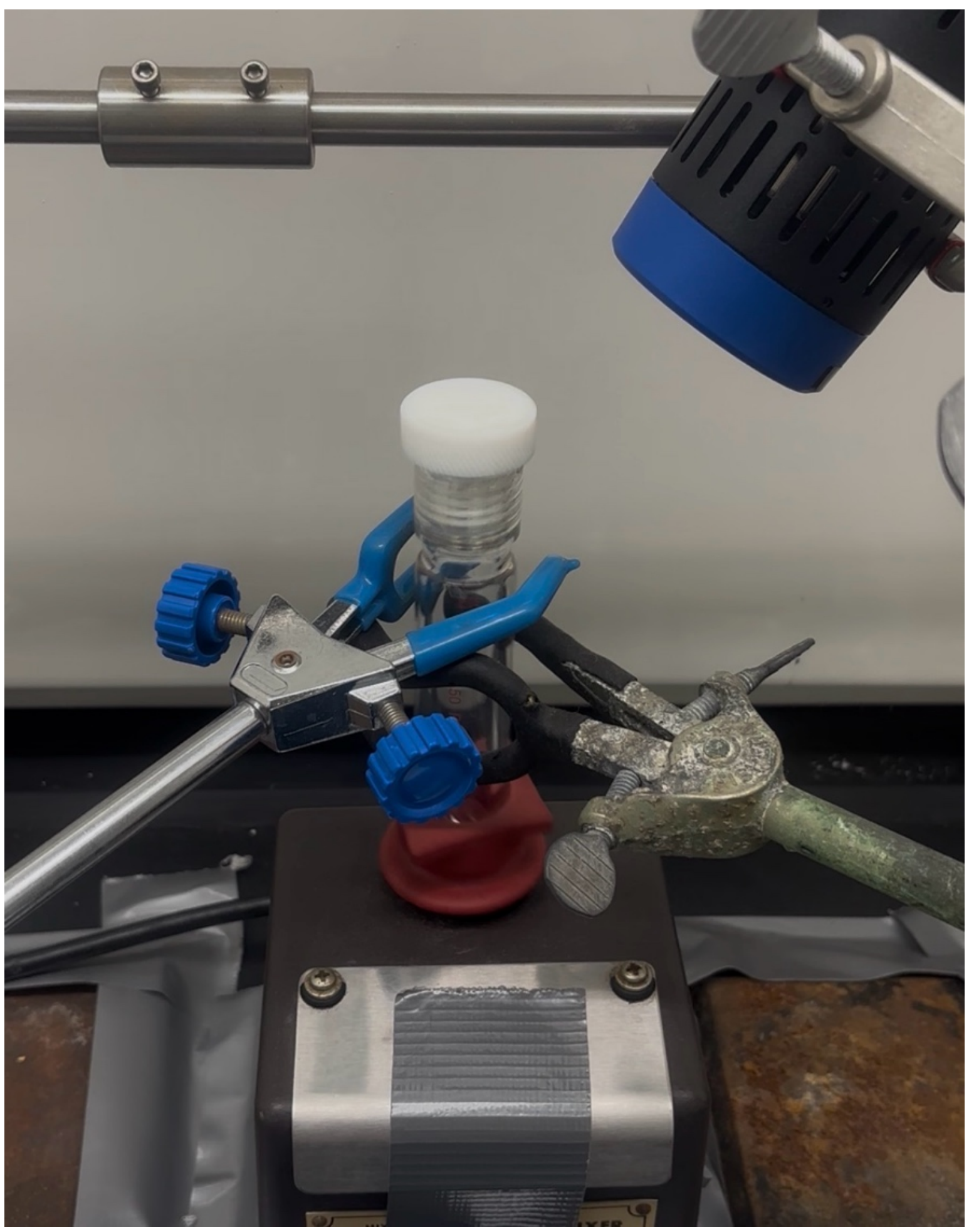

Figure S1.1: Photograph of reaction setup showing the sealed Ace glass 15 mL pressure tube, (center) vortex mixer, (bottom) and Kesel PR160L-456 UV-LED (top right) used in the reaction.

Molecular modeling

Electrostatic potential (ESP) maps were generated using density functional theory (DFT) calculations in Spartan with the ωB97X-D hybrid functional and the cc-pVTZ basis set,[8,9] starting from crystallographic coordinates. Molecular coordinates were obtained from the SCXRD data. Prior to calculating the ESP maps, geometrical optimization of the single crystal structures was performed by density functional theory calculations at the B3LYP/6-31G(d) level, where non-hydrogen atom coordinates were fixed, and only hydrogen atoms were optimized.

S2. Single-crystal X-ray diffraction data

Table S2.1: Crystallographic parameters for (**pbe**)·(**4-nvp**) and 2(**pbe**)·(**HT-pncb**).

**Compound**	(**pbe**)·(**4-nvp**)	2(**pbe**)·(**HT-pncb**)
Formula	$C_{29}H_{22}BNO_2$	$C_{29}H_{22}BNO_2$
fw [g/mol]	427.28	427.28
T [K]	100(2)	100.0(2)
Crystal System	triclinic	triclinic
Space Group	*P*-1	*P*-1
a [Å]	9.9139(9)	9.8480(6)
b [Å]	10.0410(9)	10.2044(7)
c [Å]	12.0544(11)	12.0701(6)
α [°]	94.787(3)	97.361(4)
β [°]	103.252(3)	105.352(4)
γ [°]	109.168(3)	104.675(5)
Volume [$Å^3$]	1086.69(17)	1106.47(12)
Z	2	2
ρ_{calc} [g/cm^3]	1.306	1.282
μ [mm^{-1}]	0.084	0.079
F(000)	448	448
Crystal Size [mm^3]	0.12 × 0.1 × 0.04	0.29 × 0.209 × 0.069
Radiation	synchrotron (λ = 0.7288)	Mo Kα (λ = 0.71073)
2Θ range for data collection [°]	3.614 to 68.344	4.496 to 50
Index ranges	-15 ≤ h ≤ 15, -15 ≤ k ≤ 15, -18 ≤ l ≤ 18	-11 ≤ h ≤ 11, -12 ≤ k ≤ 12, -14 ≤ l ≤ 14
Reflections collected	40745	12615
Independent reflections	8286 [R_{int} = 0.0376, R_{sigma} = 0.0302]	3872 [R_{int} = 0.0365, R_{sigma} = 0.0586]
Data/restraint/parameters	8286/0/298	3872/0/298
Goodness-of-fit on F^2	1.058	0.948
Final R indexes [I>=2σ (I)]	R_1 = 0.0440, wR_2 = 0.1190	R_1 = 0.0443, wR_2 = 0.0989
Final R indexes [all data]	R_1 = 0.0536, wR_2 = 0.1265	R_1 = 0.0829, wR_2 = 0.1112
CDCC Identification Code	2560286	2560285

Table S2.2: Intermolecular contacts in (**pbe**)·(**4-nvp**) and 2(**pbe**)·(**HT-pncb**) crystals.

Crystal	Interaction	*d*(π···π) [Å]	*d*(C-H···π) [Å]	Symmetry code
(**pbe**)·(**4-nvp**)	Cg1[a]···Pg1[b]	3.669	-	-X,1-Y,1-Z
	Cg2[c]···Cg1[a]	3.771		1-X, 2-Y,1-Z
	C25-H···Cg3[d]	-	2.909	-X,1-Y,1-Z
	C10-H··· Pg1[b]	-	3.463	X, -1+Y,Z
	C27-H··· Cg4[e]	-	2.966	-1+X, -Y,-1+Z
2(**pbe**)·(**HT-pncb**)	Cg1[a]···Pg1[a]	3.661	-	-
	Cg2[c]···Cg1[a]	3.667	-	2-X, -Y,1-Z
	C25-H···Cg3[d]	-	2.866	-
	C9-H···Pg1[b]	-	3.906	X, -1+Y, Z
	C27-H··· Cg4[e]	-	2.692	-1+X, -Y,-1+Z

[a]Cg1 = C24-C29, [b]Pg1 = N1, C13-C17, [c]Cg2=C21-C24,C29, [d]Cg3 = C7-C12, [e]Cg4 = C1-C6

S3. Powder X-ray diffraction data

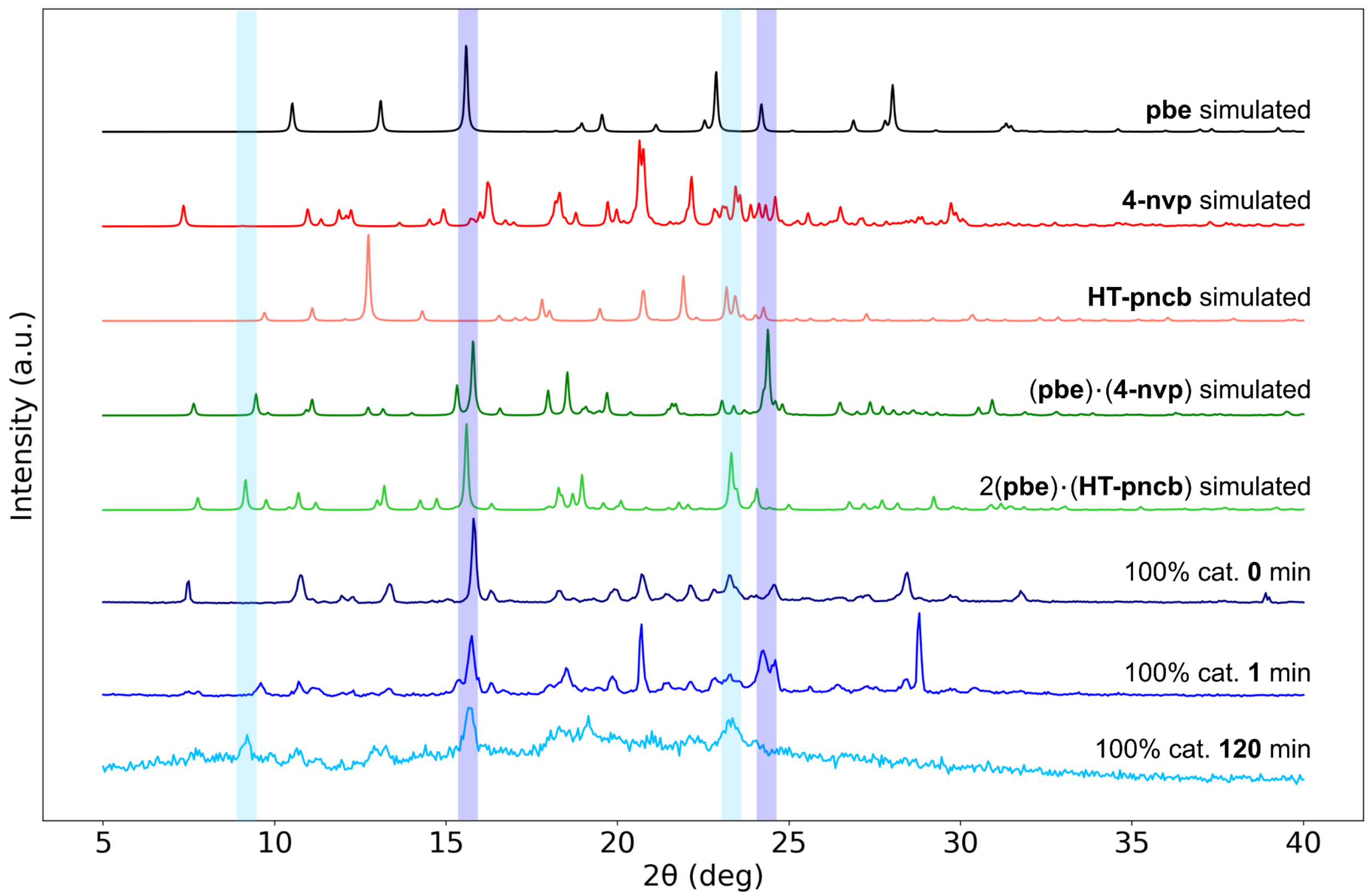

Figure S3.1: Expanded PXRD monitoring of the solid-state photomechanochemical reaction (100% mol cat. **pbe**). The experimental traces were recorded at 0, 1, and 120 minutes of concurrent UV-LED exposure and milling. The 0-minute time point represents the pre-irradiation state, recorded after 10 seconds of preliminary grinding. Simulated patterns (top five traces) of SCXRD data of the individual starting materials (**pbe**, **4-nvp**), the unbound photoproduct (**HT-pncb**), and the bound adducts 2(**pbe**)·(**HT-pncb**) and (**pbe**)·(**4-nvp**) are provided for comparison. Shaded regions highlight key diagnostic peaks used to track the reaction progress.

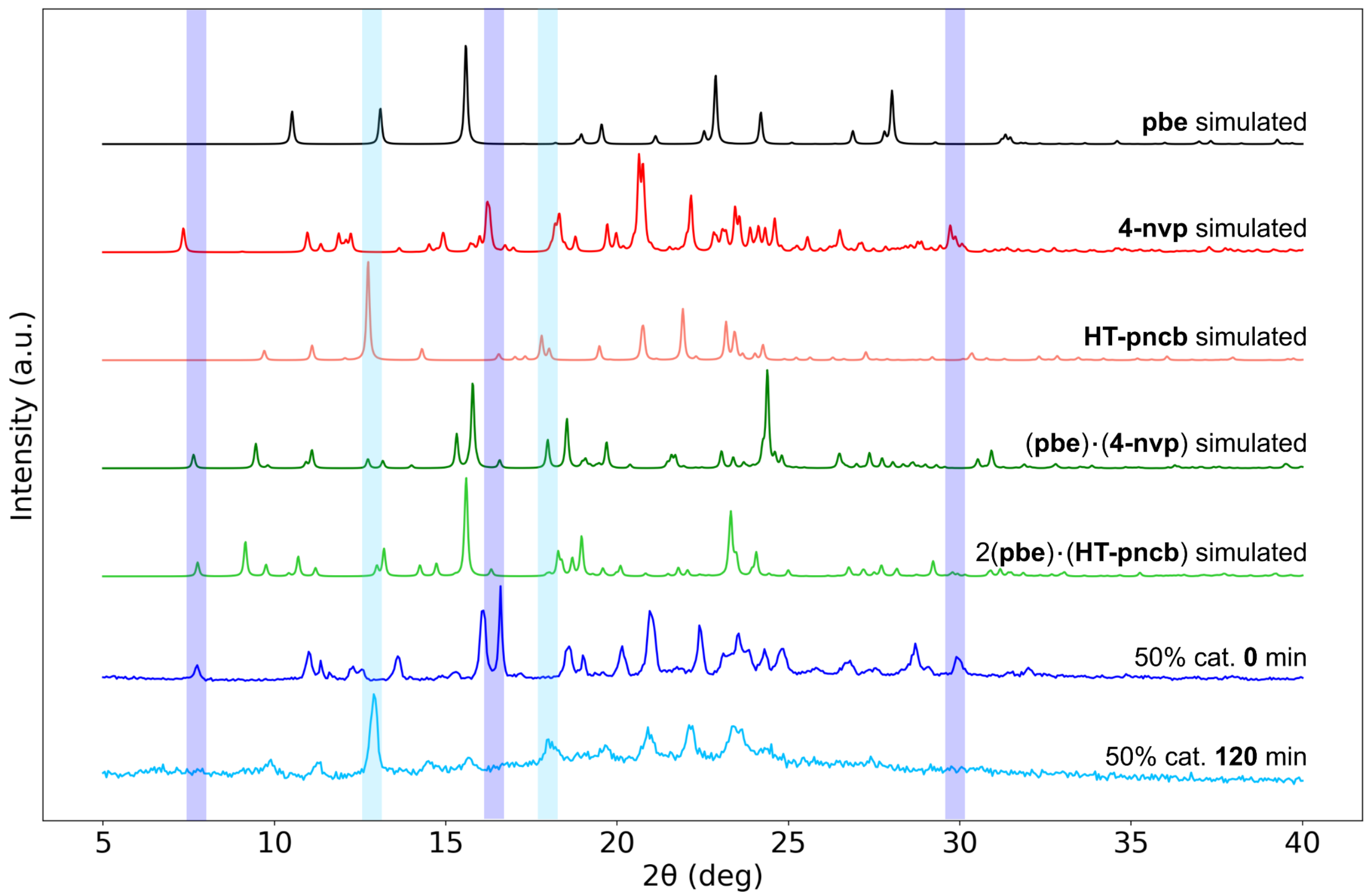

Figure S3.2: Expanded PXRD monitoring of the solid-state photomechanochemical reaction (50% mol cat. **pbe**). The experimental traces were recorded at 0, 1, and 120 minutes of concurrent UV-LED exposure and milling. The 0-minute time point represents the pre-irradiation state, recorded after 10 seconds of preliminary grinding. Simulated patterns (top five traces) of SCXRD data of the individual starting materials (**pbe**, **4-nvp**), the unbound photoproduct (**HT-pncb**), and the bound adducts 2(**pbe**)·(**HT-pncb**) and (**pbe**)·(**4-nvp**) are provided for comparison. Shaded regions highlight key diagnostic peaks used to track the reaction progress.

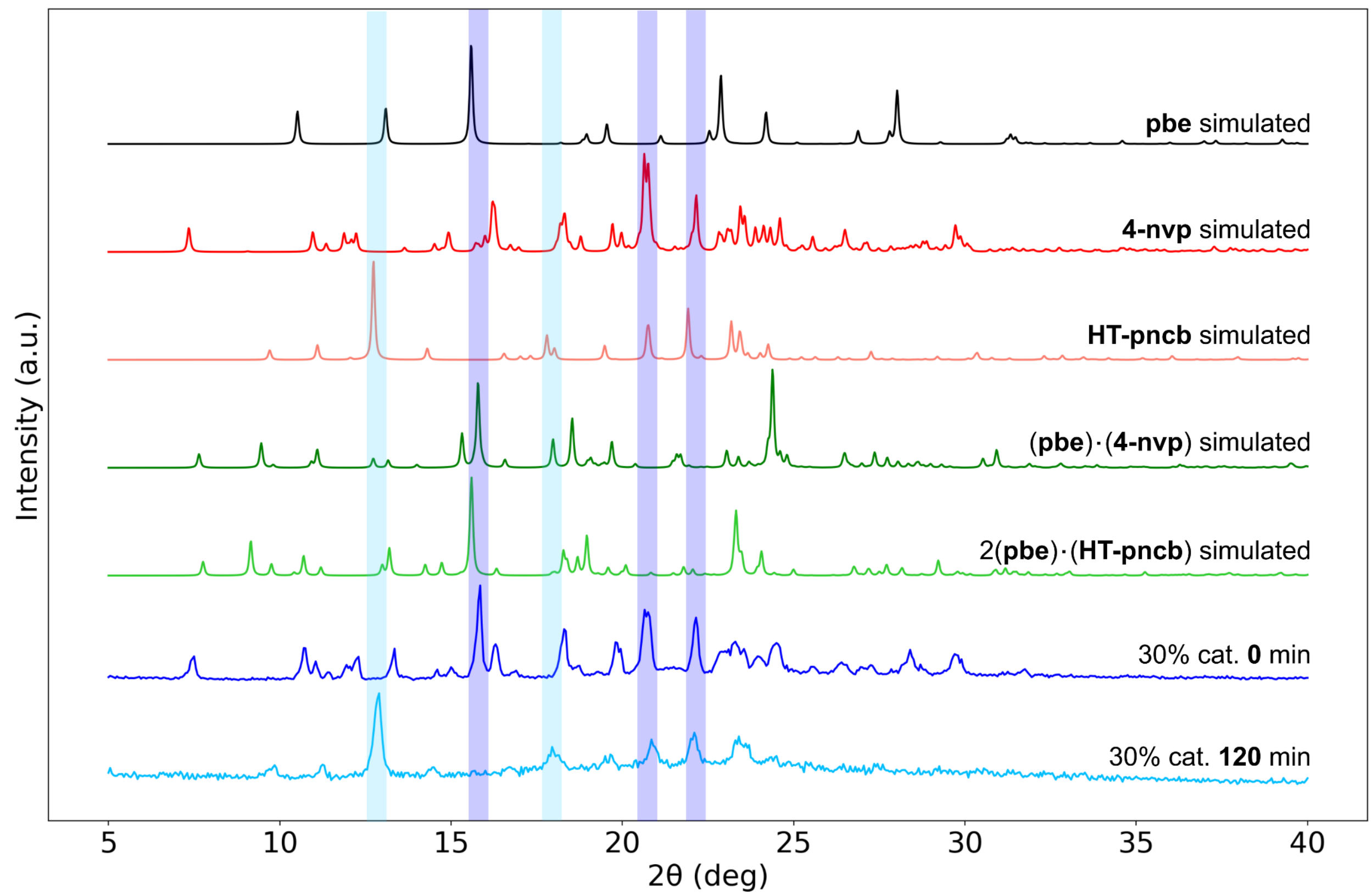

Figure S3.3: Expanded PXRD monitoring of the solid-state photomechanochemical reaction (30% mol cat. **pbe**). The experimental traces were recorded at 0, 1, and 120 minutes of concurrent UV-LED exposure and milling. The 0-minute time point represents the pre-irradiation state, recorded after 10 seconds of preliminary grinding. Simulated patterns (top five traces) of SCXRD data of the individual starting materials (**pbe**, **4-nvp**), the unbound photoproduct (**HT-pncb**), and the bound adducts 2(**pbe**)·(**HT-pncb**) and (**pbe**)·(**4-nvp**) are provided for comparison. Shaded regions highlight key diagnostic peaks used to track the reaction progress.

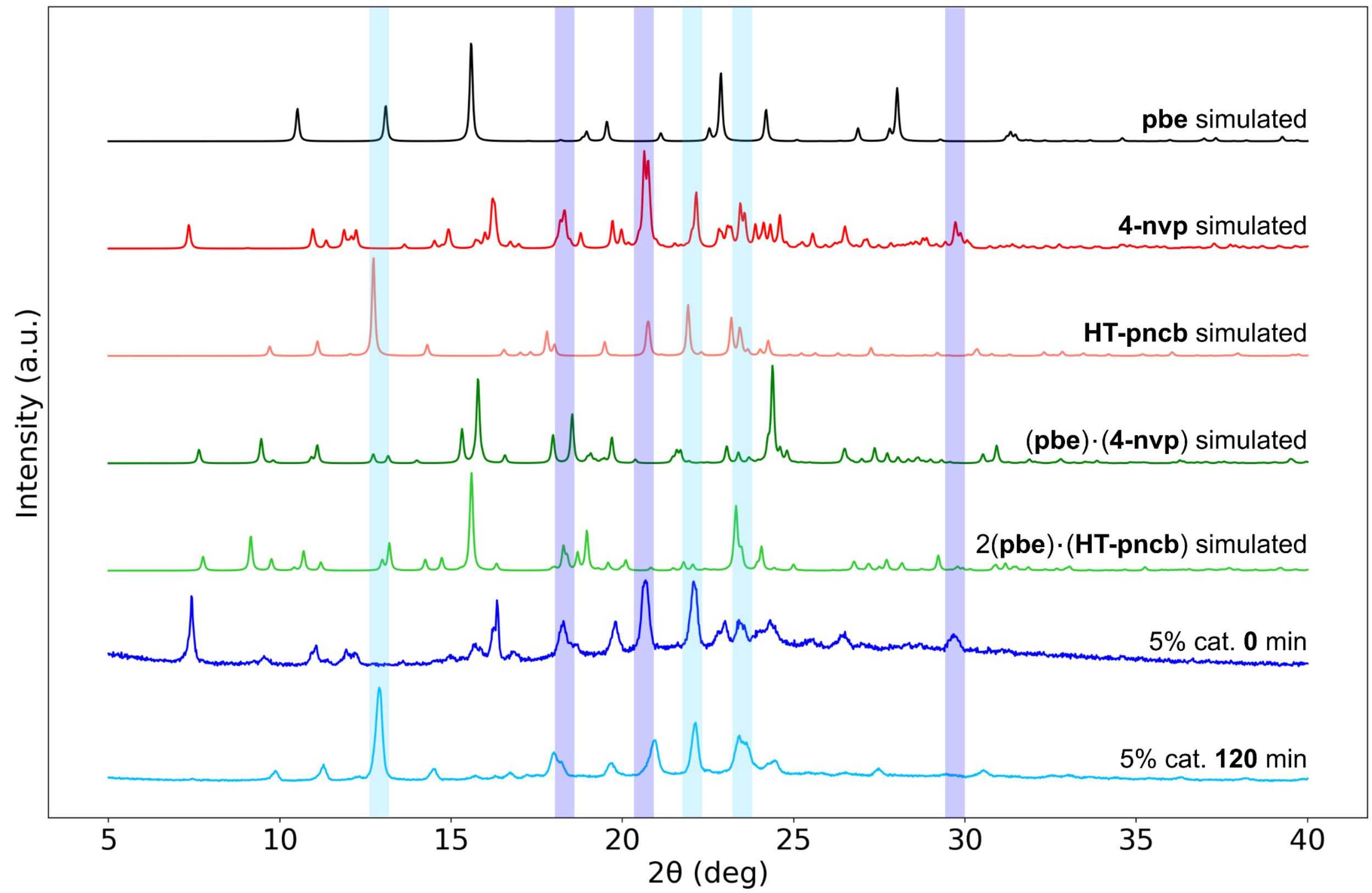

Figure S3.4: Expanded PXRD monitoring of the solid-state photomechanochemical reaction (5% mol cat. **pbe**). The experimental traces were recorded at 0, 1, and 120 minutes of concurrent UV-LED exposure and milling. The 0-minute time point represents the pre-irradiation state, recorded after 10 seconds of preliminary grinding. Simulated patterns (top five traces) of SCXRD data of the individual starting materials (**pbe**, **4-nvp**), the unbound photoproduct (**HT-pncb**), and the bound adducts 2(**pbe**)·(**HT-pncb**) and (**pbe**)·(**4-nvp**) are provided for comparison. Shaded regions highlight key diagnostic peaks used to track the reaction progress.

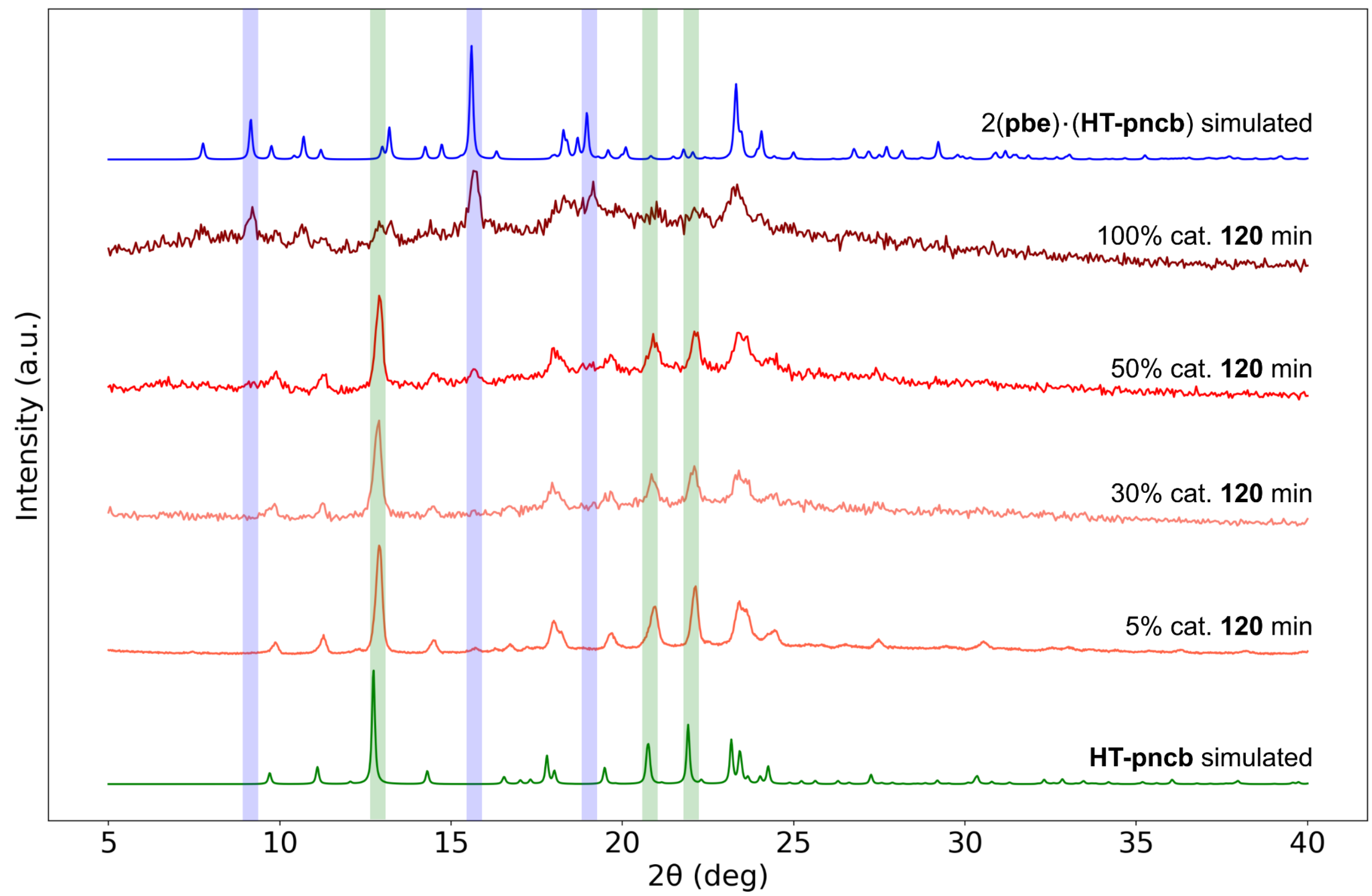

Figure S3.5: Expanded PXRD monitoring of the solid-state photomechanochemical reactions (100%, 50%, 30%, and 5% mol cat. **pbe**). The experimental traces recorded at 120 minutes of UV-LED exposure (the middle four traces) are compared with each other. The 120-minute time point represents the final state, recorded after 120 minutes of combined irradiation and grinding. The simulated patterns (top and bottom traces) of the bound 2(**pbe**)·(**HT-pncb**) and unbound **HT-pncb** are provided for comparison. Shaded regions highlight the different majority species present at different **pbe** loads.

S4. NMR spectroscopy data

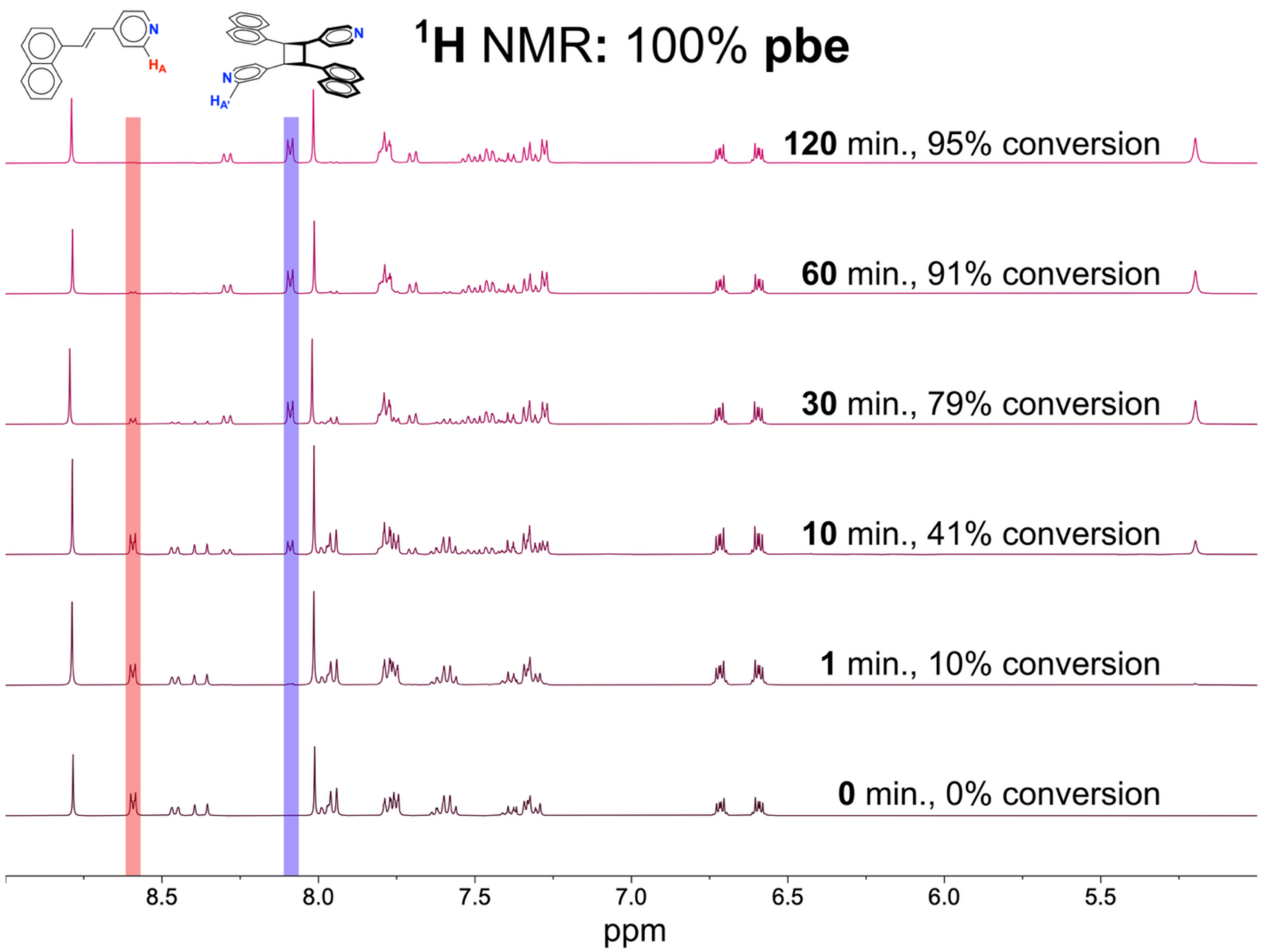

Figure S4.1: Expanded ^{1}H NMR (400 MHz, DMSO-d_6) spectra taken at 0, 1, 10, 30, 60, and 120 minutes of reaction time for monitoring (100% mol cat. **pbe**). Conversion yields are calculated as the ratio of the integration value of $H_{A'}$ (blue) to the sum of the integration values of H_A (red) and $H_{A'}$ (blue). Note that B←N adducts hydrolyze into phenylboronic acid, catechol, and free *N*-donor in DMSO-d_6.

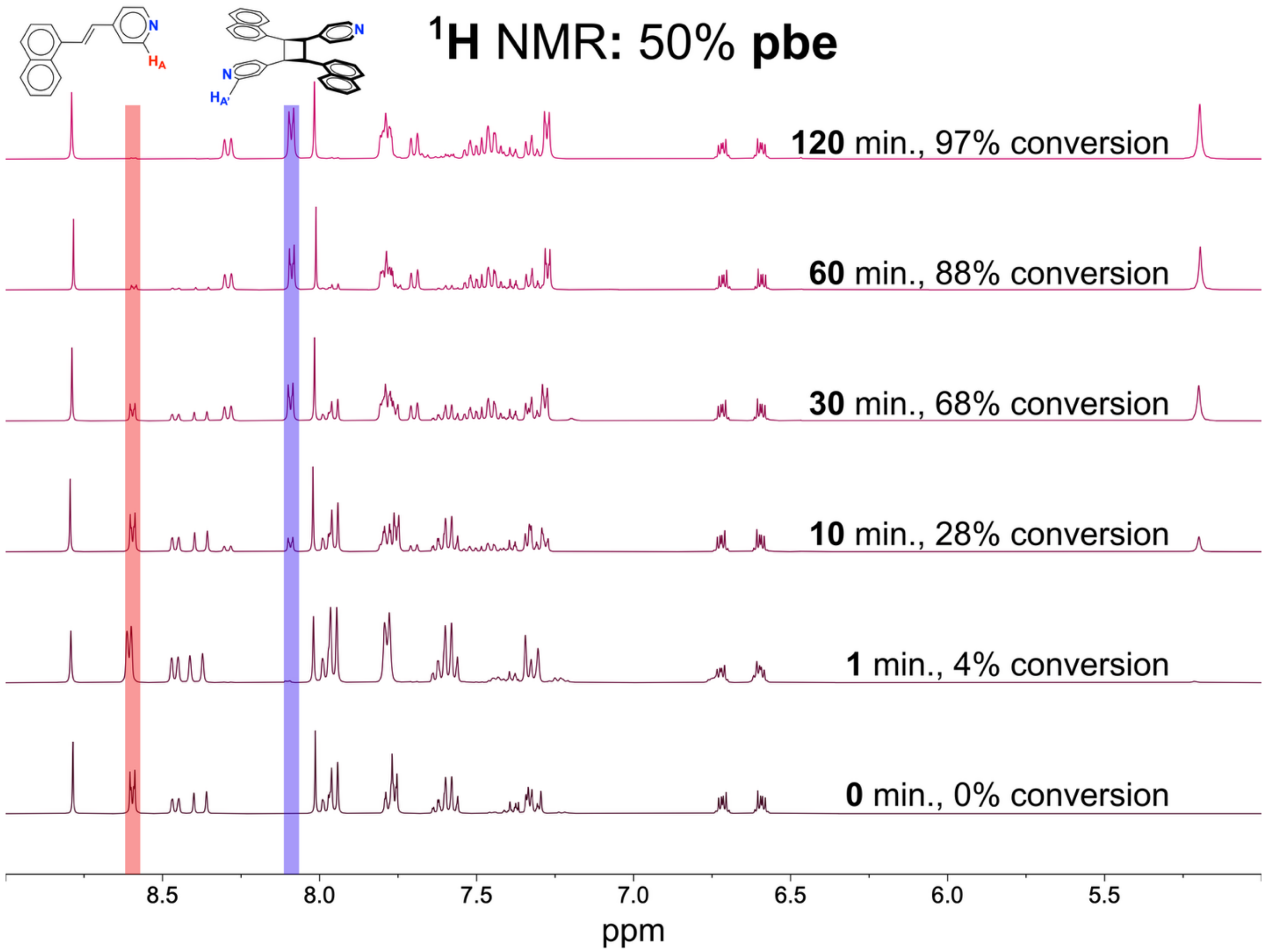

Figure S4.2: Expanded ^{1}H NMR (400 MHz, DMSO-d_6) spectra taken at 0, 1, 10, 30, 60, and 120 minutes of reaction time for monitoring (50% mol cat. **pbe**). Conversion yields are calculated as the ratio of the integration value of $H_{A'}$ (blue) to the sum of the integration values of H_A (red) and $H_{A'}$ (blue). Note that B←N adducts hydrolyze into phenylboronic acid, catechol, and free *N*-donor in DMSO-d_6.

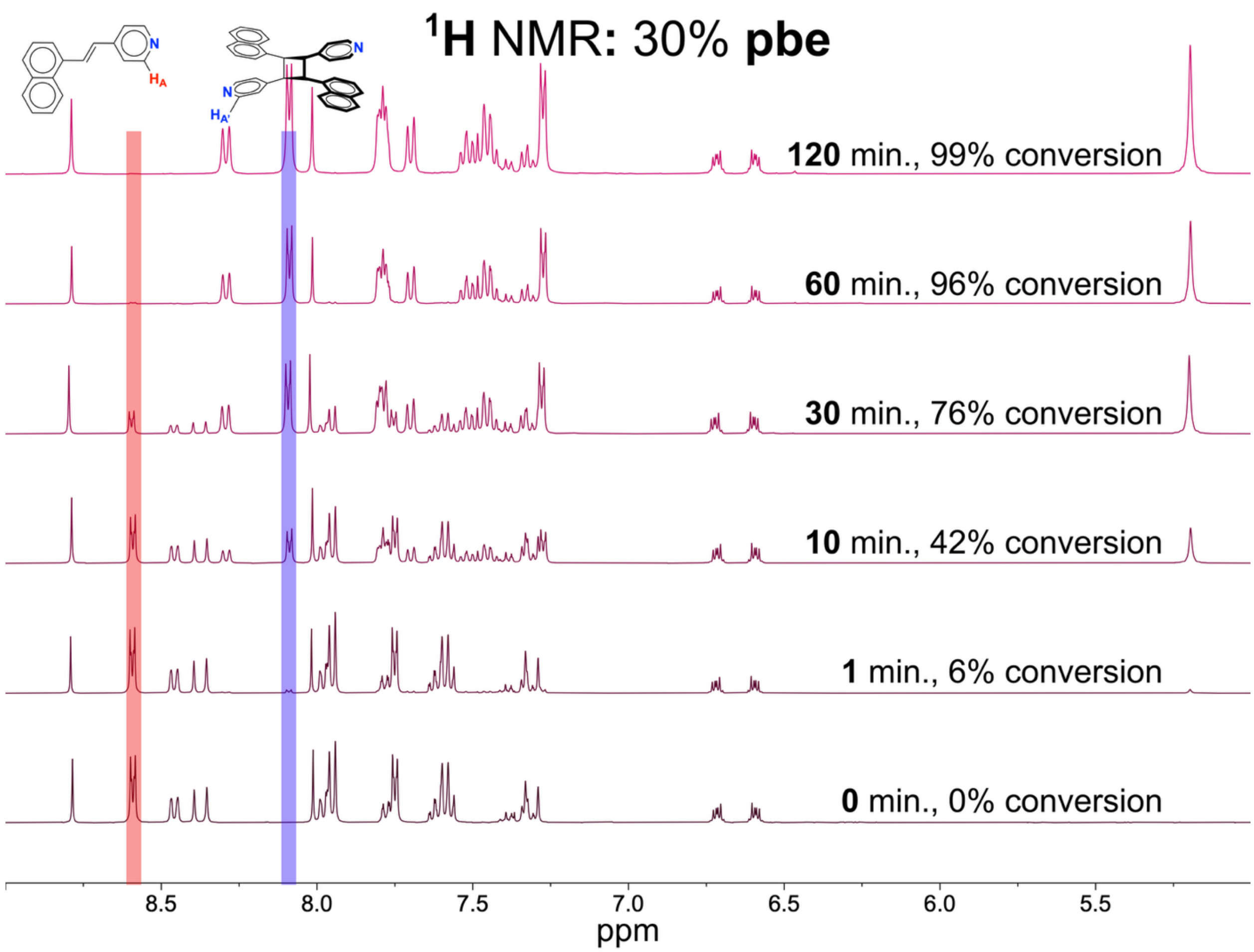

Figure S4.3: Expanded ^{1}H NMR (400 MHz, DMSO-d_6) spectra taken at 0, 1, 10, 30, 60, and 120 minutes of reaction time for monitoring (30% mol cat. **pbe**). Conversion yields are calculated as the ratio of the integration value of $H_{A'}$ (blue) to the sum of the integration values of H_A (red) and $H_{A'}$. Note that B←N adducts hydrolyze into phenylboronic acid, catechol, and free *N*-donor in DMSO-d_6.

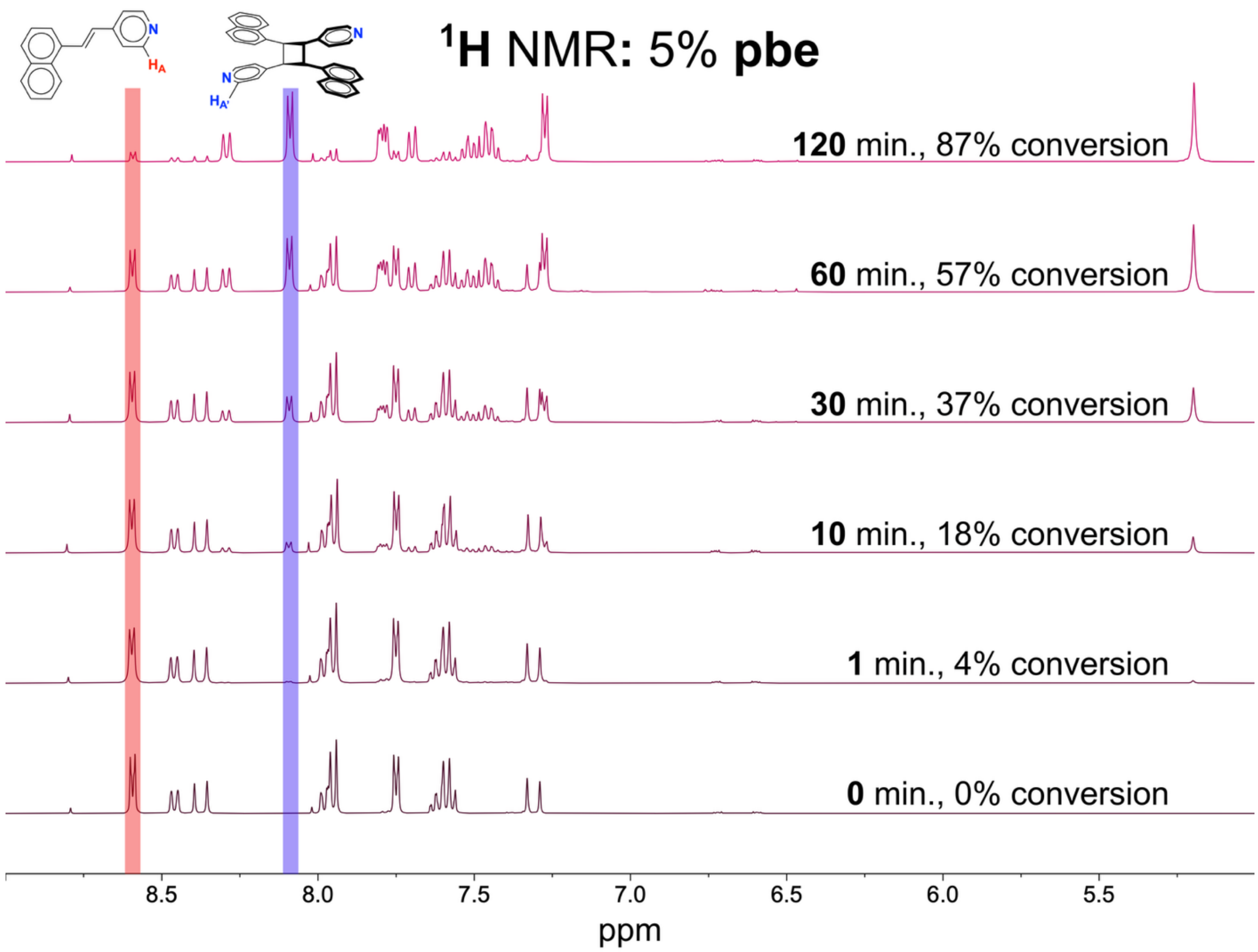

Figure S4.4: Expanded ^{1}H NMR (400 MHz, DMSO-d_6) spectra taken at 0, 1, 10, 30, 60, and 120 minutes of reaction time for monitoring (5% mol cat. **pbe**). Conversion yields are calculated as the ratio of the integration value of $H_{A'}$ (blue) to the sum of the integration values of H_A (red) and $H_{A'}$ (blue). Note that B←N adducts hydrolyze into phenylboronic acid, catechol, and free *N*-donor in DMSO-d_6.

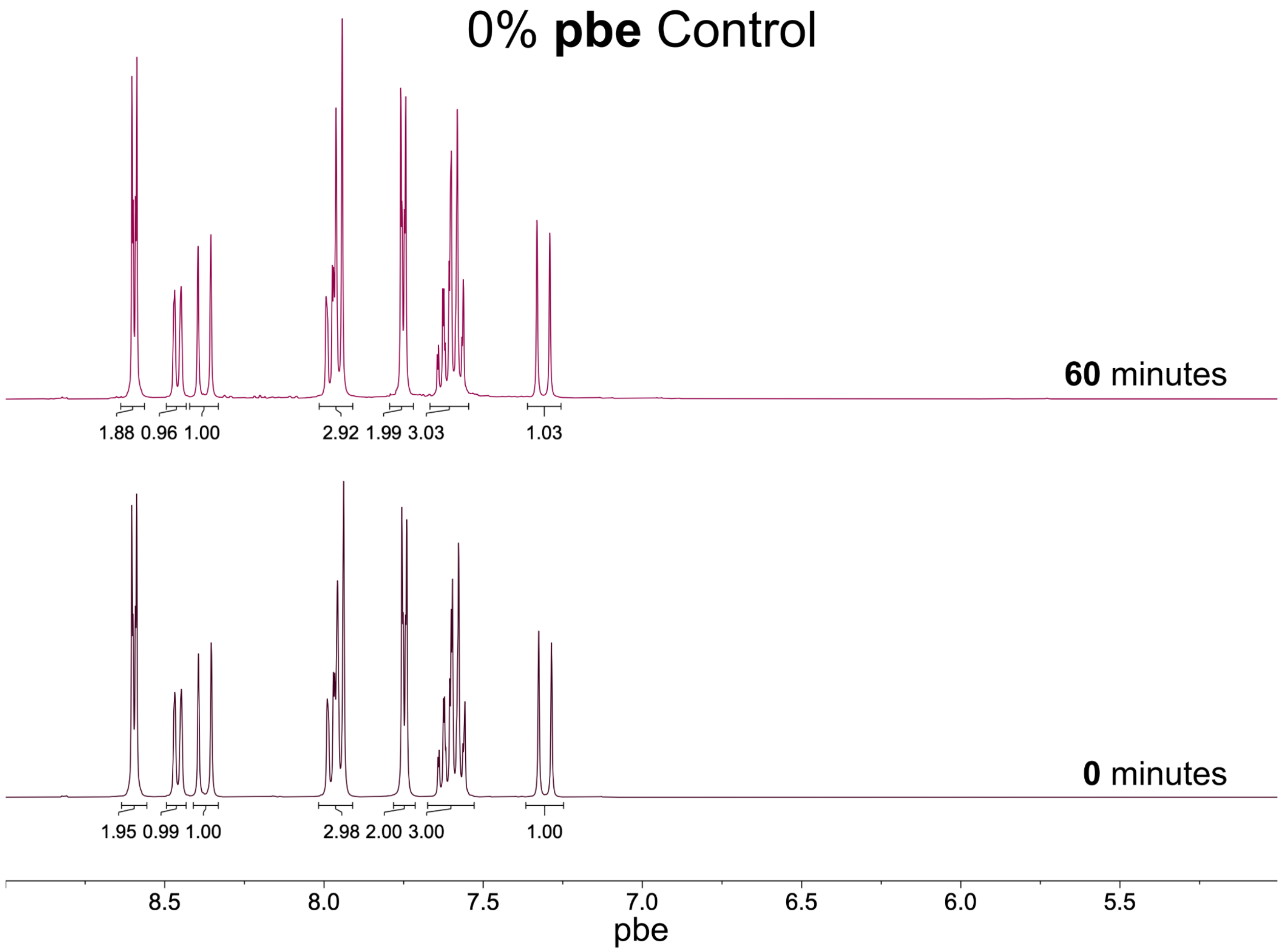

Figure S4.5: Expanded ^{1}H NMR (400 MHz, DMSO-d_6) spectra taken at 0 and 60 minutes of combined UV exposure and grinding time for monitoring (5% mol cat. **pbe**).

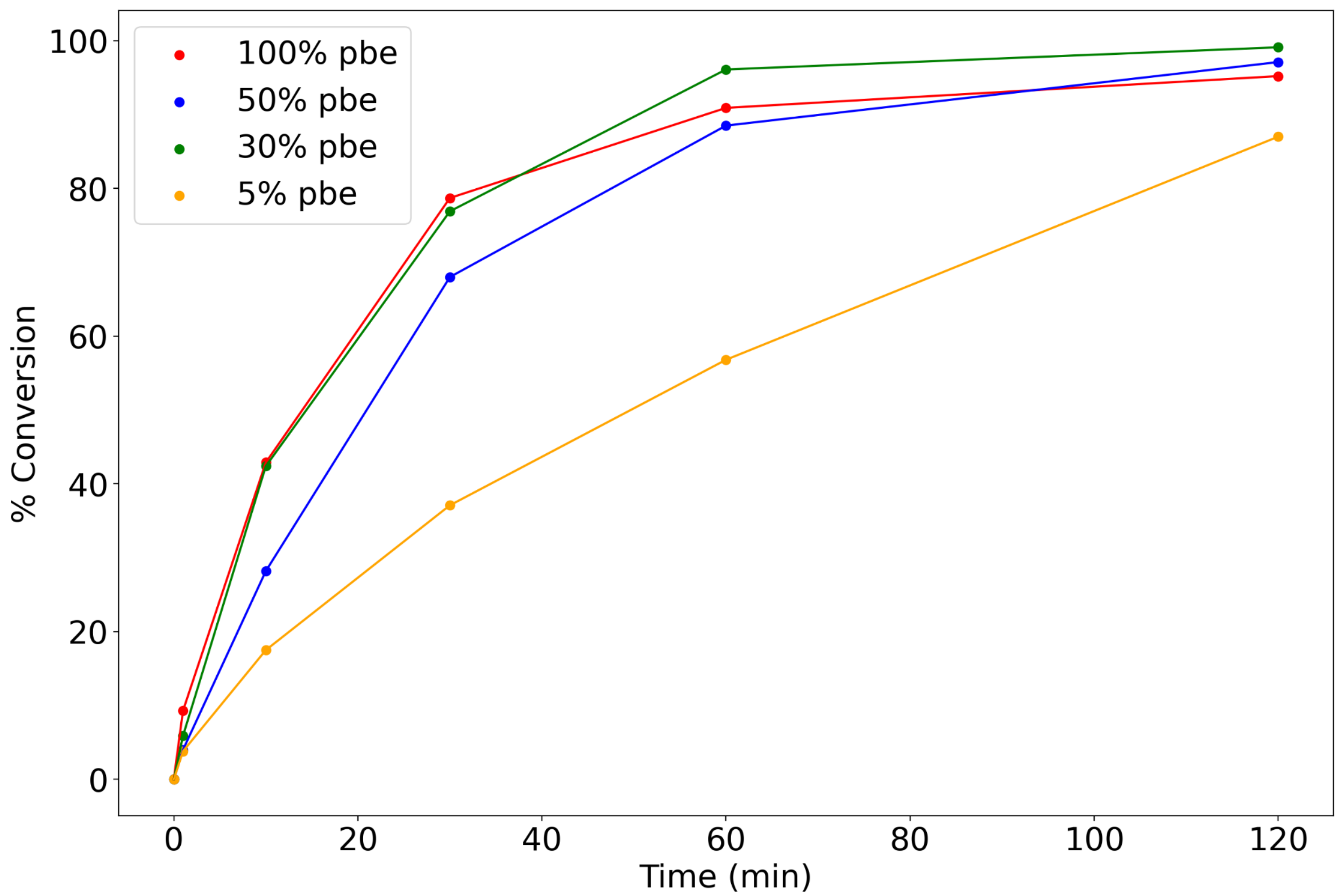

Figure S4.6: Reaction yields from different catalytic loads of **pbe** (100% red, 50% blue, 30% green, 5% yellow) plotted as the ratio of $H_{A'}$ to the sum of H_A and $H_{A'}$ (using integration values from ^{1}H NMR) vs time.

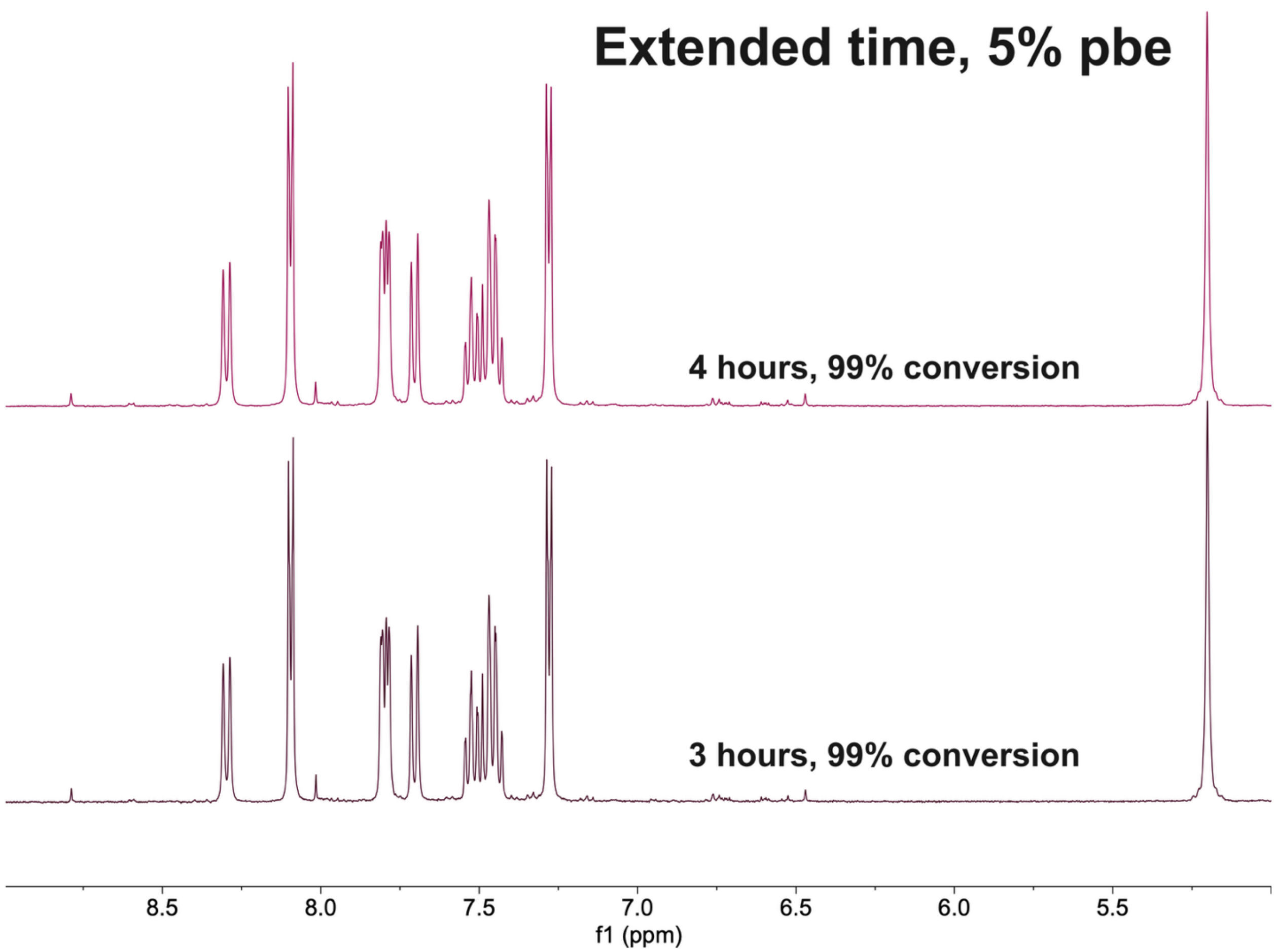

Figure S4.7: Expanded ^{1}H NMR (400 MHz, DMSO-d_6) spectra taken at 180 min (3 h) and 240 min (4 h) of combined UV exposure and grinding, both showing quantitative yields. Note that B←N adducts hydrolyze into phenylboronic acid, catechol, and free *N*-donor in DMSO-d_6.

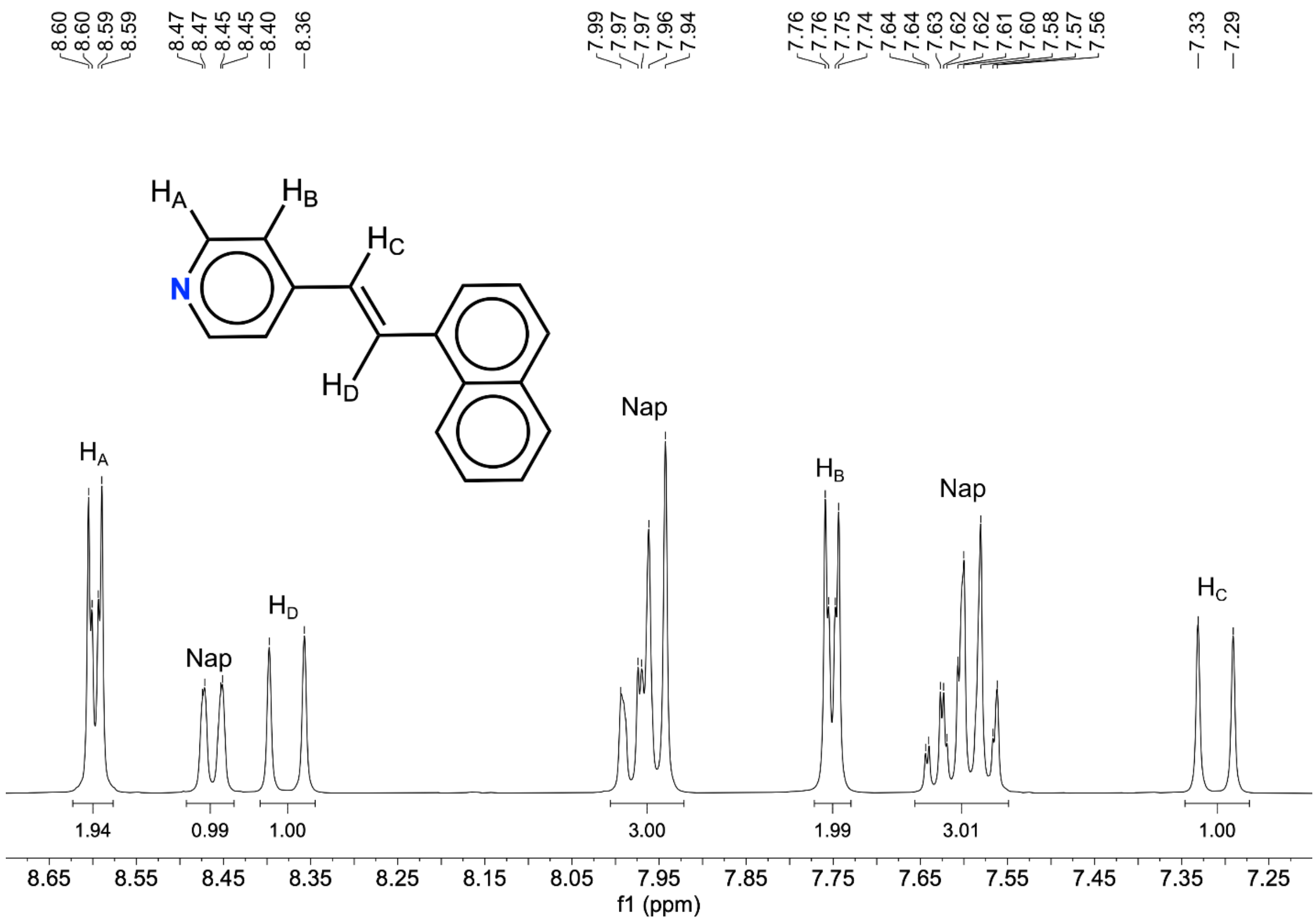

Figure S4.8: ^{1}H NMR (400 MHz, DMSO-d_6) spectrum of **4-nvp**.

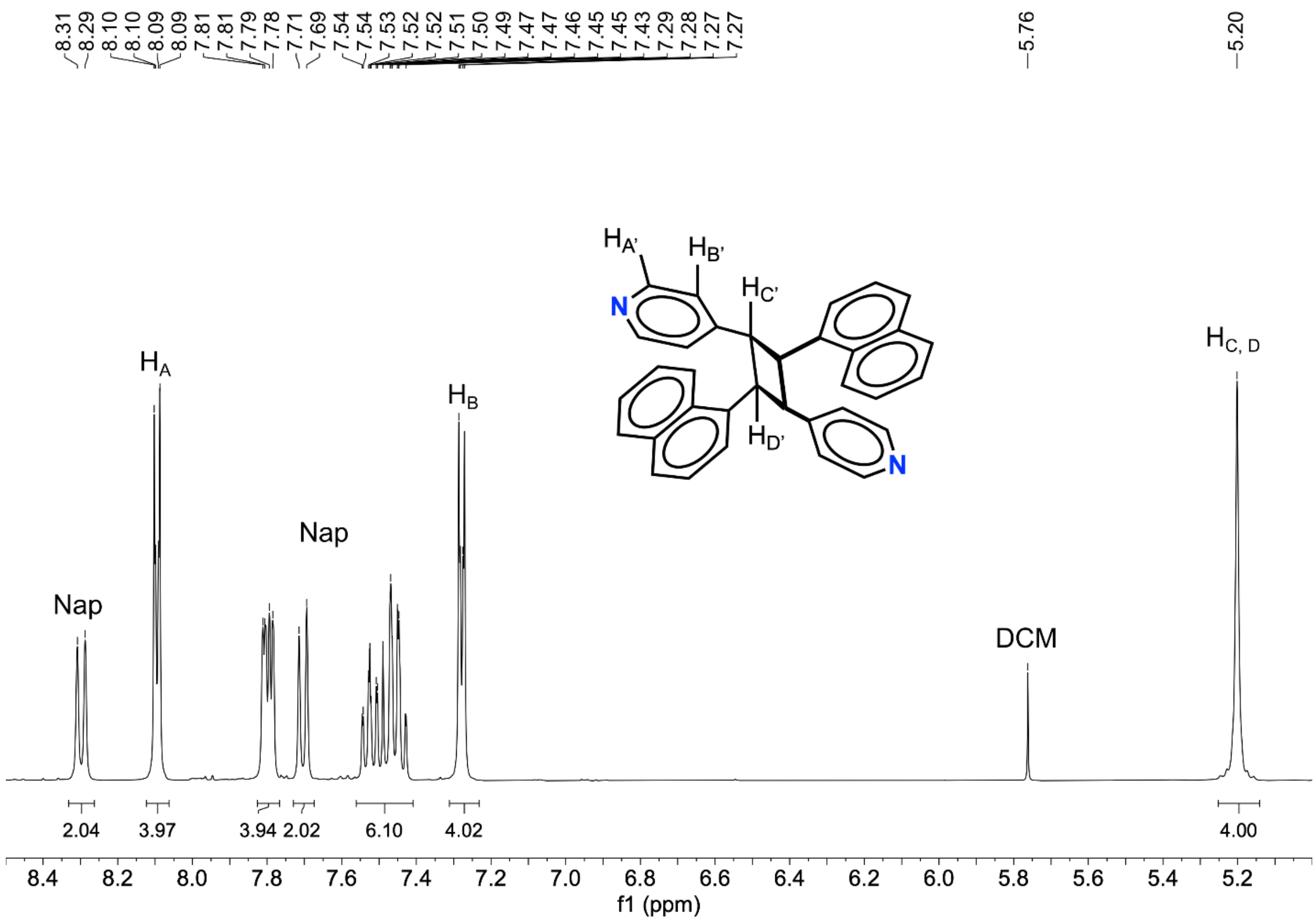

Figure S4.9: ^{1}H NMR (400 MHz, DMSO-d_6) spectrum of **HT-pncb**.

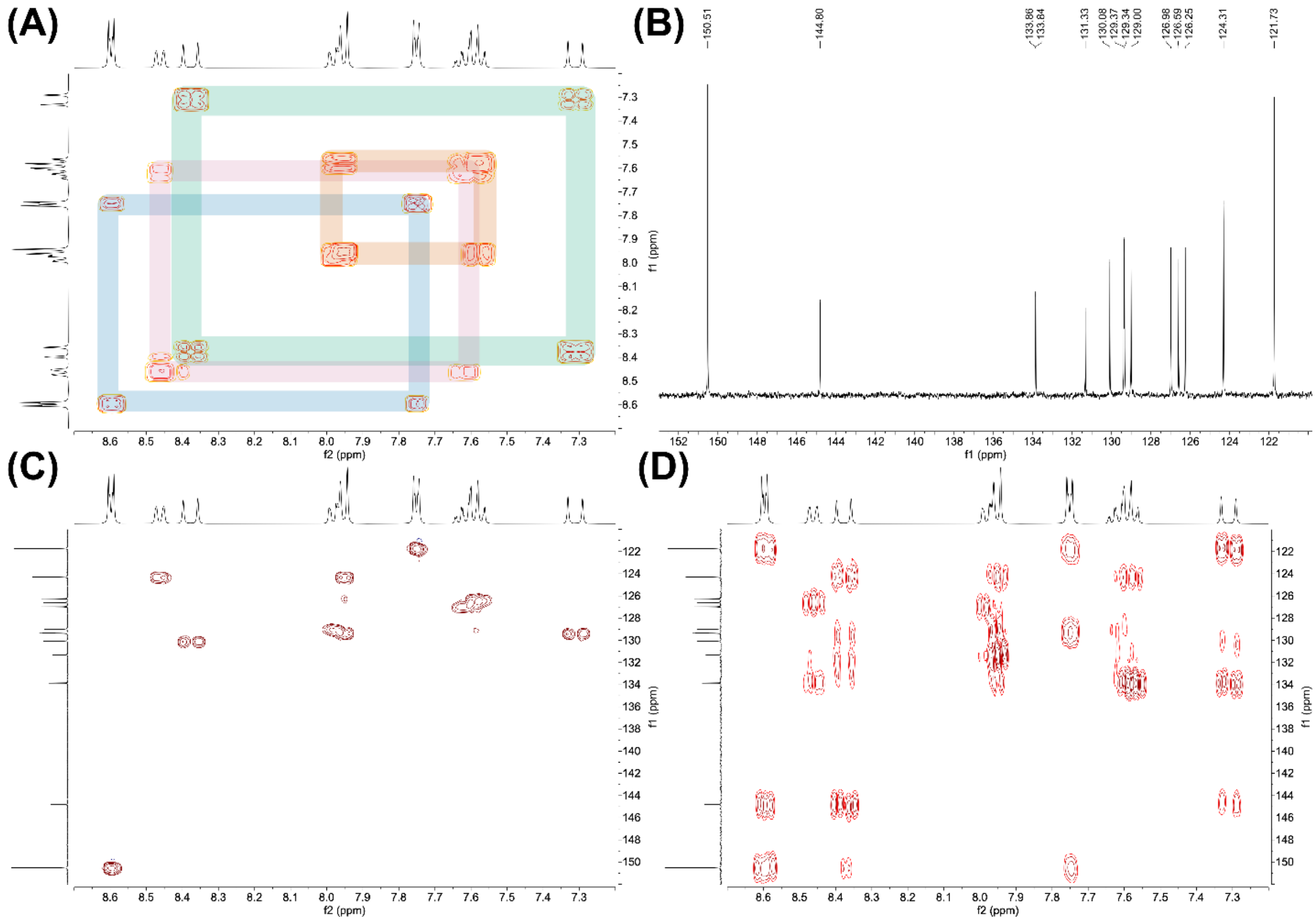

Figure S4.10: Additional NMR (400 MHz, DMSO-d_6) characterization for **4-nvp**. (A) ^1H-^1H COSY (400 MHz, DMSO-d_6), (B) ^{13}C NMR (101 MHz, DMSO-d_6), (C) ^{13}C-^1H HSQC, (D) ^{13}C-^1H HMBC.

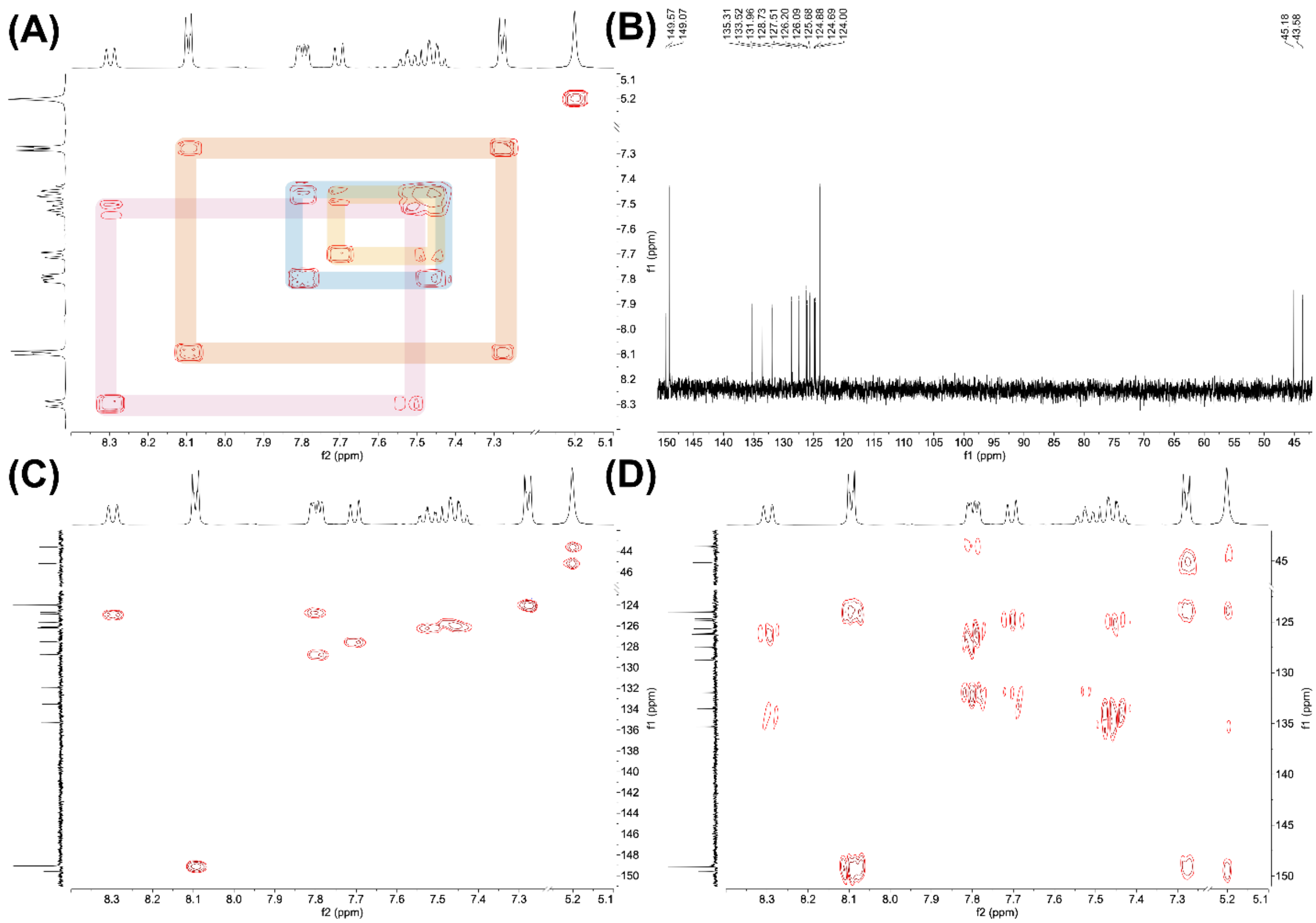

Figure S4.11: Additional NMR (400 MHz, DMSO-d_6) characterization for **HT-pncb**. (A) ^1H-^1H COSY (400 MHz, DMSO-d_6), (B) ^{13}C NMR (101 MHz, DMSO-d_6), (C) ^{13}C-^1H HSQC, (D) ^{13}C-^1H HMBC.

S5. Molecular modeling data

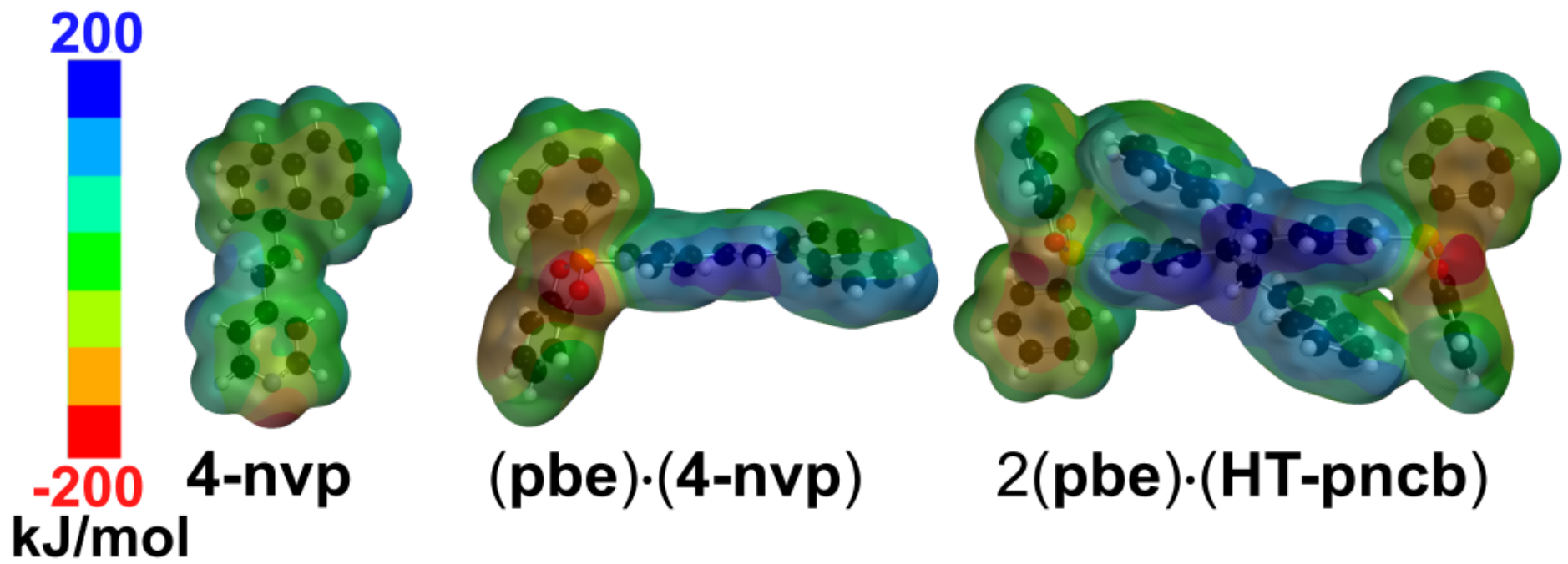

Figure S5.1: Electrostatic potential (ESP) maps of **(pbe)**·**(4-nvp)** and 2(**pbe**)·(**HT-pncb**) crystals.

S6. Fourier-transform infrared data

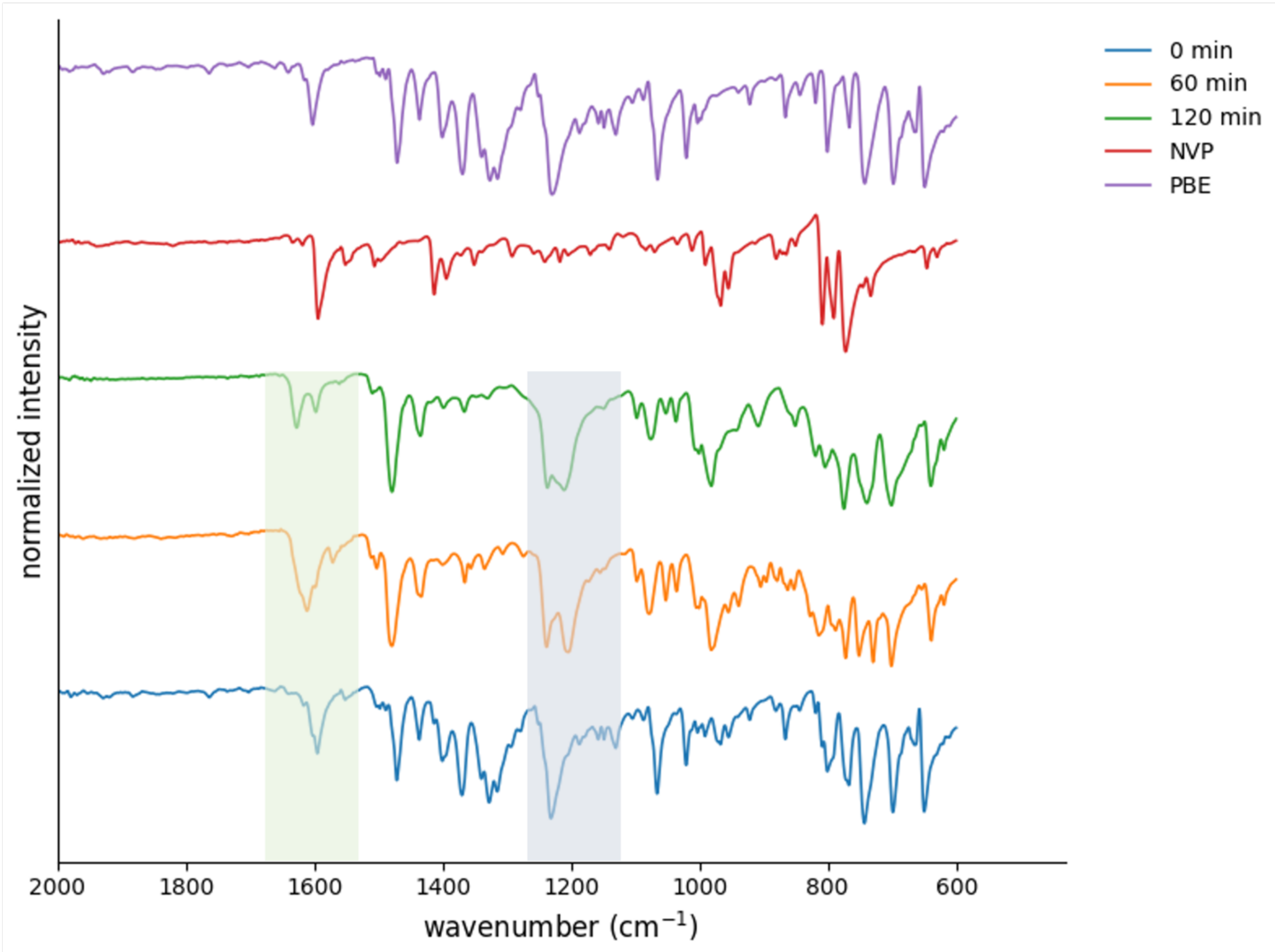

Figure S6.1: FT-IR spectra of starting materials **pbe** and **4-nvp**, and of powdered samples of equimolar amounts (100% mol cat. **pbe**) taken at 0, 60, and 120 minutes of combined UV exposure and grinding. The spectra show changes in the fingerprint region consistent with conversion of **4-nvp** to the cyclobutane photoproduct.

S7. Thermogravimetric analysis data

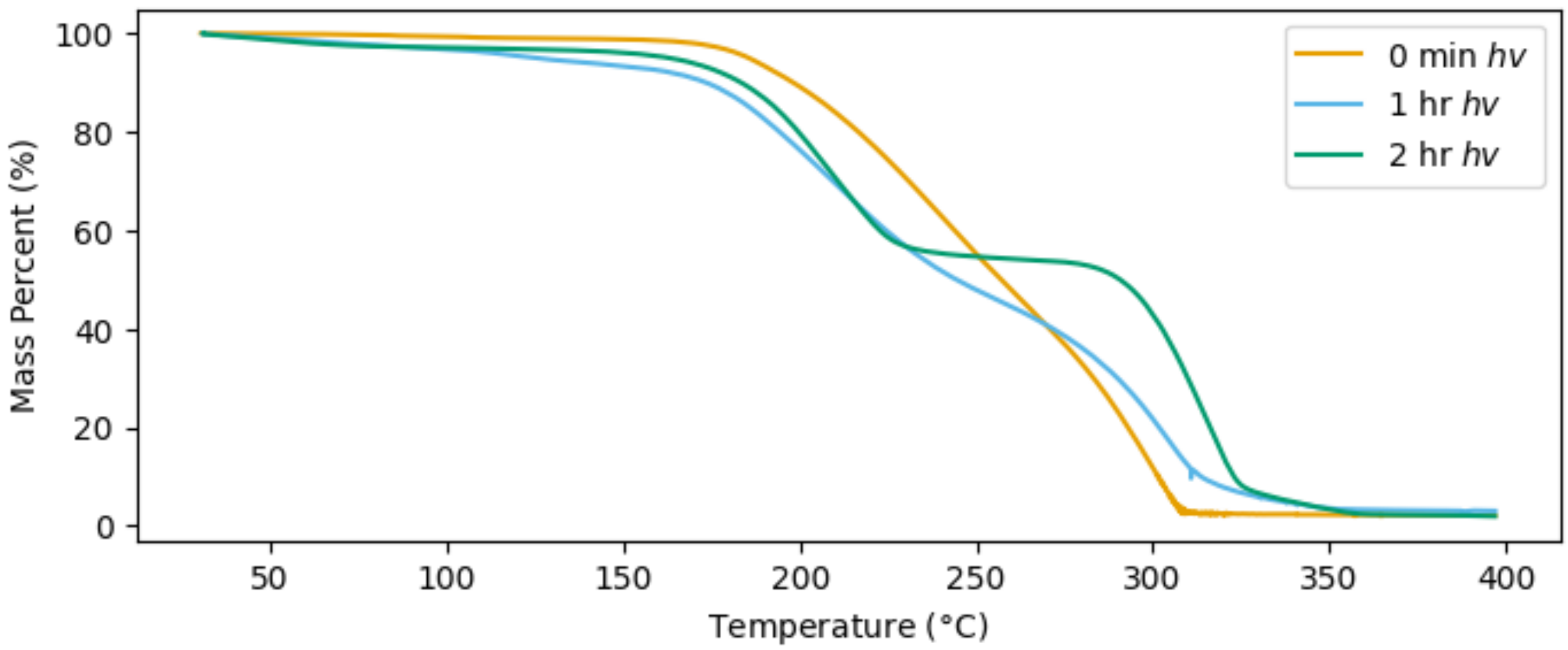

Figure S7.1: TGA traces of powdered samples taken at 0, 60, and 120 minutes of combined UV exposure and grinding. For the sample taken at 120 min, the total mass loss at 270 °C is 46.2%, which corresponds to the mass of **pbe**.

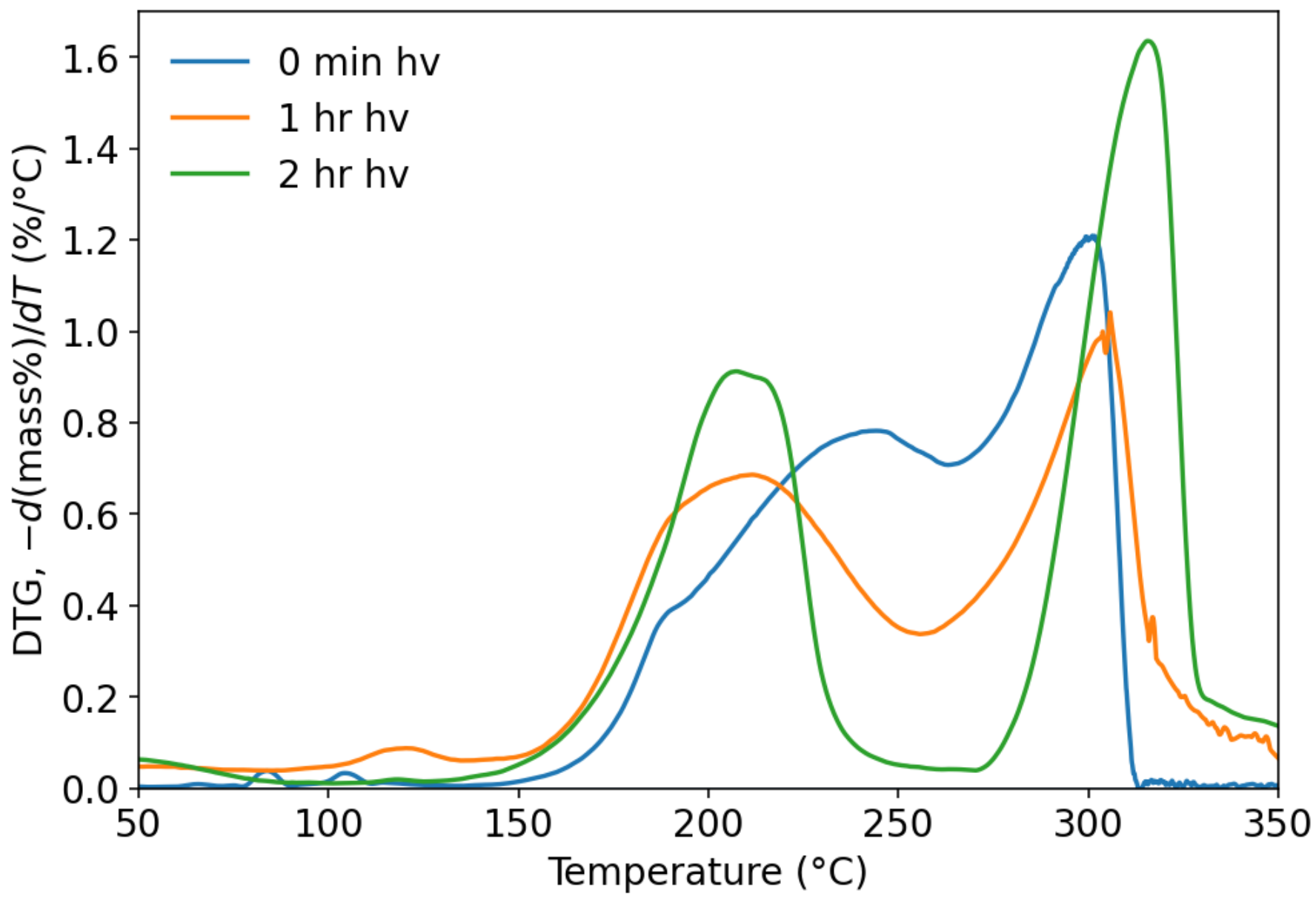

Figure S7.2: Derivative thermogravimetric (DTG) traces of powdered samples taken at 0, 60, and 120 minutes of combined UV exposure and grinding showing changes in the temperatures and relative rates of the principal mass-loss events.

Thermogravimetric and derivative thermogravimetric analyses (TGA and DTG) showed a distinct change in thermal behavior after 120 min, with two resolved mass-loss events, including an initial 46.2% loss that closely matches the calculated **pbe** content (46%) of 2(**pbe**)·(**HT-pncb**).

References

(1) Bruker, U. *APEX3 V2019. 1–0, SAINT V8. 40A*; Bruker AXS Inc.: Madison (WI), USA, 2019.

(2) SAINT, S. Area-Detector Integration Program. *V6* **1995**, *22*, 1997–2001.

(3) Blessing, R. H. An Empirical Correction for Absorption Anisotropy. *Acta Crystallogr. A* **1995**, *51* (1), 33–38. https://doi.org/10.1107/S0108767394005726.

(4) Kissel, L.; Pratt, R. H. Corrections to Tabulated Anomalous-Scattering Factors. *Acta Crystallogr. A* **1990**, *46* (3), 170–175. https://doi.org/10.1107/S0108767389010718.

(5) Farrugia, L. J. WinGX Suite for Small-Molecule Single-Crystal Crystallography. *J. Appl. Crystallogr.* **1999**, *32* (4), 837–838. https://doi.org/10.1107/S0021889899006020.

(6) Sheldrick, G. M. SHELXT – Integrated Space-Group and Crystal-Structure Determination. *Acta Crystallogr. Sect. Found. Adv.* **2015**, *71* (1), 3–8. https://doi.org/10.1107/S2053273314026370.

(7) Sheldrick, G. M. Crystal Structure Refinement with SHELXL. *Acta Crystallogr. Sect. C Struct. Chem.* **2015**, *71* (1), 3–8. https://doi.org/10.1107/S2053229614024218.

(8) Chai, J.-D.; Head-Gordon, M. Long-Range Corrected Hybrid Density Functionals with Damped Atom–Atom Dispersion Corrections. *Phys. Chem. Chem. Phys.* **2008**, *10* (44), 6615–6620. https://doi.org/10.1039/B810189B.

(9) Kang, Y. K.; Park, H. S. Assessment of CCSD(T), MP2, DFT-D, CBS-QB3, and G4(MP2) Methods for Conformational Study of Alanine and Proline Dipeptides. *Chem. Phys. Lett.* **2014**, *600*, 112–117. https://doi.org/10.1016/j.cplett.2014.03.067.
